# The Experiences of Parents and Infants Using a Home-Based Art Intervention Aimed at Improving Wellbeing and Connectedness in Their Relationship

**DOI:** 10.3389/fpsyg.2022.732562

**Published:** 2022-05-17

**Authors:** Victoria Gray Armstrong, Josephine Ross

**Affiliations:** Psychology, School of Social Sciences, University of Dundee, Dundee, United Kingdom

**Keywords:** art, attachment, connection, wellbeing, dyads, parent, infant

## Abstract

During the period of COVID-19 restrictions, we offered vulnerable families with 0 to 3 year old children boxes of art resources and guided creative activities to do together at home. This paper explores families’ experiences of this intervention, highlighting their perceptions of change in wellbeing and attachment. There is a developing case for the social benefits of art, including the impact of arts on mental health and on the wellbeing of children. However, we know that social factors impact upon arts participation, and existing inequalities and mental health difficulties have been exacerbated in the context of the pandemic. This project aimed to adapt to restrictions, to provide a meaningful remote intervention, supporting parent-infant dyads to have positive interactions through art making. We sought to explore the benefits of this intervention for infants and parents with a view to understanding more about the psychological benefits of art participation and about ways to engage families into art making, as well as thinking about how best we can evidence these kinds of arts in health interventions. Preliminary findings showed promising outcomes from the art boxes and this paper brings together the full results, primarily based on interviews with sixteen parents and four referrers alongside collected feedback. We highlight potential mechanisms for change within the intervention and detail the perceived impact of the art boxes in supporting attachment. Parents felt that the art-boxes facilitated changes in their own wellbeing that would make them more available to connection, and recognised changes for babies that reflected their increased capacity to mentalise about their child. Importantly, there were also concrete changes for the dyad that represented improved connection, such as more playful time together and increased shared attention and eye contact. Our observations suggest that the quality of the parent-infant relationship benefited from home-based art intervention, and we speculate about the potential efficacy of this approach beyond the pandemic.

## Introduction

Art at the Start is a research project embedded within a public arts venue, aiming to explore the benefits of early art making for families with 0 to 3 year old children. We approach this aim from various angles, including art therapy sessions for parents and infants at risk of attachment difficulties, and public sessions seeking to increase families’ use of art with very young children. During the COVID-19 restrictions, we maintained contact with the families referred to us for attachment difficulties. We achieved this by providing ‘art boxes’, containing materials and activity guides for families to make art together at home. The current paper explores the benefits of this home-based art intervention for infants and parents, with a view to understanding more about the psychological benefits of art participation, about ways to engage families into art making, and how best we can evidence arts in health interventions.

### The Psychological Benefits of Arts Based Interventions and Their Social Context

There is increased awareness of the social benefits of art participation (Fancourt and Finn, 2019), including the impact of arts on mental health (Davies et al., 2016; Jensen and Bonde, 2018) and on the wellbeing of children (Bungay and Vella-Burrows, 2013; Mak and Fancourt, 2019). Art venues therefore have an important role to play in wellbeing for individuals, families and communities (Crossick and Kaszynska, 2016). However, social factors, such as socio-economic status, deprivations, disabilities, existing health conditions, ethnicity and family background, impact upon arts participation (Matthews et al., 2016; Fancourt and Steptoe, 2019; Mak et al., 2020). As a result, arts venues must rise to the challenge to facilitate engagement for all communities (Silverman, 2010).

In our locality (Dundee, Scotland) families can face multiple disadvantages, with more than a third of areas among the most deprived in the country (SIMD, 2020). Difficulties impacting upon children include a higher percentage of first-time mothers under age 19 than the national average, and more parents living with long-term physical or mental health issues. An estimated 30.1% of children live in households that experience both low income and material deprivation (Dundee City Council, 2019). The pandemic can be seen to have aggravated existing inequalities (Blundell et al., 2020), including the early experiences of children (Papworth et al., 2021b). Research has found a significant impact on children’s rights from the COVID-19 measures, including poverty, food insecurity and equality of digital access (Treaner, 2020). A 2020 report into digital exclusion in Scotland gave an estimate of 800,000 people facing digital inequalities, a main driver of which was poverty (Halliday, 2020). This is particularly crucial for the arts sector given that many galleries and museums tried to remain connected to their audiences in 2020 by creating online content, such as children’s art ideas. This means digital exclusion may have exaggerated existing inequalities in children’s and families’ access to the arts at this time. Preconceptions about the need for art resources and materials in the home may also have put families off from accessing online content related to art making. In addition to physical resources, the use of online arts content requires less tangible resources within the family unit; significantly, an adult carer needs to have capacity (both in terms of time and mental wellbeing) to make such activities available for children in the home.

An estimated 10–20% of mothers develop mental health difficulties during pregnancy and in the year after birth (Bauer et al., 2014). Post-natal depression is perhaps the most researched and recognised mental health difficulty affecting new mothers, with universal screening in place in the United Kingdom (Field, 2017). However, there are clear barriers to accessing support (Goodman, 2009). There is also growing research into the high prevalence and under-recognition of post-partum anxiety (Zappas et al., 2020) and the risk of post-traumatic stress disorder (Harrison et al., 2021). Evidence of similar mental health difficulties for new fathers, and the impact of this on their children, is also growing (Paulson and Bazemore, 2010; Kahn, 2017). The impact of the pandemic in exacerbating parental mental health difficulties has been demonstrated with prompt research showing increased rates of post-natal depression (Wu et al., 2020) and increased depression and anxiety in mothers of younger children and pregnant women (Cameron et al., 2020; Lebel et al., 2020). In particular, Gassman-Pines et al. (2020) found the impact was greater for those who were already vulnerable. A review of maternal mental health during the pandemic (Papworth et al., 2021a) found an increased risk of mental health difficulties, including stress and anxiety caused by giving birth over this period and concerns about their infants. They also found that services for perinatal mental health were stretched pre-pandemic and increasingly inadequate during the pandemic. At the same time voluntary sector, community and informal support was greatly reduced. Importantly, the quality of early attachment relationships is known to be impacted where a parent has poor mental health (Cummings and Cicchetti, 1990) with both depression and anxiety associated with impaired parent-infant bonding (Moehler et al., 2006; Davies et al., 2021).

Parent-infant relationships are likely to have similarly been impacted by the pandemic. A report into the experiences of 0–2 s during the pandemic (Reed and Parish, 2021), though highlighting that there may have been some benefits through increased time with both parents in the home, is clear that there are risk factors in the reduction of direct services. They also identify potential harms from lack of social interactions, exposure to trauma, material deprivation and the increased stress upon parents resulting in less responsive parenting. A report from a survey of the parents of babies during the pandemic (Saunders and Hogg, 2020) found that a quarter were concerned about their relationship to their baby and a third felt that their interactions had changed. The attachment relationship with their primary caregivers, and the responsiveness of care which they receive within that, will be crucial for infants’ emotional wellbeing (Benoit, 2004). From an experience of responsive care, infants develop their own sense of self and develop social and emotional skills such as the capacity to regulate or to relate to others (Schore, 2001; Landry et al., 2006; Barlow et al., 2010). Secure attachments will support their development and wellbeing throughout childhood (Sroufe, 1996; Warren et al., 1997; Belsky, 2001) whilst insecurity results in a heightened risk of later social problems and behavioural difficulties (Belsky and Fearon, 2002). Concerningly, studies found that, even before the pandemic, between a third and a half of children were insecurely attached (van IJzendoorn et al., 1999).

Fortunately, early attachments are open to change (van IJzendoorn et al., 1995) Interventions that focus on the dyads relationship and their interactions have been shown to be beneficial to attachments (Cohen et al., 2002; Baradon, 2005). Within the context of dyadic art therapy sessions, research studies have shown improvements in parental wellbeing, mental health and attachment relationships (Arroyo and Fowler, 2013; Armstrong and Howatson, 2015; Armstrong et al., 2019; Lavey-Khan and Reddick, 2020; Bruce and Hackett, 2021). A review of art therapy with this client group found that the capacity to bring dyads together into positive interactions was central (Armstrong and Ross, 2020). Benefits from shared art making in the early years has also been demonstrated in a participative art context (Starcatchers, 2014; Black et al., 2015). Art making inherently allows for intersubjective communication (Dissanayake, 2000; Gerber et al., 2018) so may be particularly suitable to support attachment. Where both parent and infant are active participants in art making, it may help to draw them together in a playful way. This has potential to benefit the wellbeing of both parent and child and can facilitate moments of the kinds of sensitive, connected experiences, which build secure attachment relationships (Meins et al., 2001; Bigelow et al., 2010).

### The Current Study

In March 2020, due to the COVID-19 public health measures and restrictions, all our face-to-face art therapy work was put on hold. We were concerned about the withdrawal of support for vulnerable families, and their relative lack of resources to participate in some of the online activities offered by ourselves and others over this time. To address this concern, we piloted an innovative method to promote the families’ engagement in joint art making by providing boxes of art materials and guided activities to do together at home. The intention was to support psychological wellbeing and encourage positive playful interaction during the pandemic restrictions. However, given the potential of the art boxes to reduce inequalities in access to the arts, we also wished to explore whether this would be a useful intervention for engaging vulnerable families beyond the pandemic. The research sits within a pragmatic paradigm as a piece of action research; seeking to create change through the research process (Greenwood and Levin, 2007) and drawing on the methodologies which best address research questions as opposed to fitting into a particular world view (Kelly and Cordeiro, 2020). This feels a good fit within Arts in Health research, where we may often be bringing together quite diverse perspectives from the humanities and medical sciences, focusing enquiry on actionable knowledge. By using rich qualitative analysis of how families experienced the pandemic, and the intervention, we aimed to gain insight into whether families perceived the art boxes to be helpful, and why. The voice of the family is important, and our approach informs debate about how best we can evidence arts in health interventions in a meaningful way. Our overarching research questions were:

1. What challenges for connection did our families (referred for attachment difficulties and/or mental health problems) face during the COVID-19 restrictions?
2. How did the ‘home art box’ intervention affect these experiences? Did parents perceive change in their own and/or their infant’s behaviours?
3. If ‘art boxes’ are perceived to create change, what might the mechanisms of this change be?

## Materials and Methods

### Participants

The art boxes were a rapid adaptation when planned supports for families were put on hold. This intervention and research had ethics approval from the University of Dundee research ethics committee (SRECPhD-033). Initially art boxes went to 40 families who had been due to commence art therapy sessions with the project but that were cancelled due to COVID-19. These families had consented to being referred for art therapy by health visitors, family nurses and voluntary services due to concerns about their attachment relationships. We reached out to all families whose sessions had been cancelled to offer them a box when it became clear the delay to sessions was going to be long term. All families who were offered a box chose to receive it. As pandemic restrictions continued, this initiative was subsequently expanded to take referrals from health visitors, family nurses (working with young parents) and three charity partners (working with ethnic minority families and families facing deprivations). Again, families provided consent for referral. Referral criteria were for parents and their infant (aged between 0 and 3 years) where there were concerns about the attachment relationship and referrers thought they would benefit from play in the home. Referral was based on the concerns of the referrers and we accepted every referral that met our inclusion criteria without screening or taking any baseline measures. This was due to the projects desire to quickly support families in the face of restrictions.

**Figure 1:**
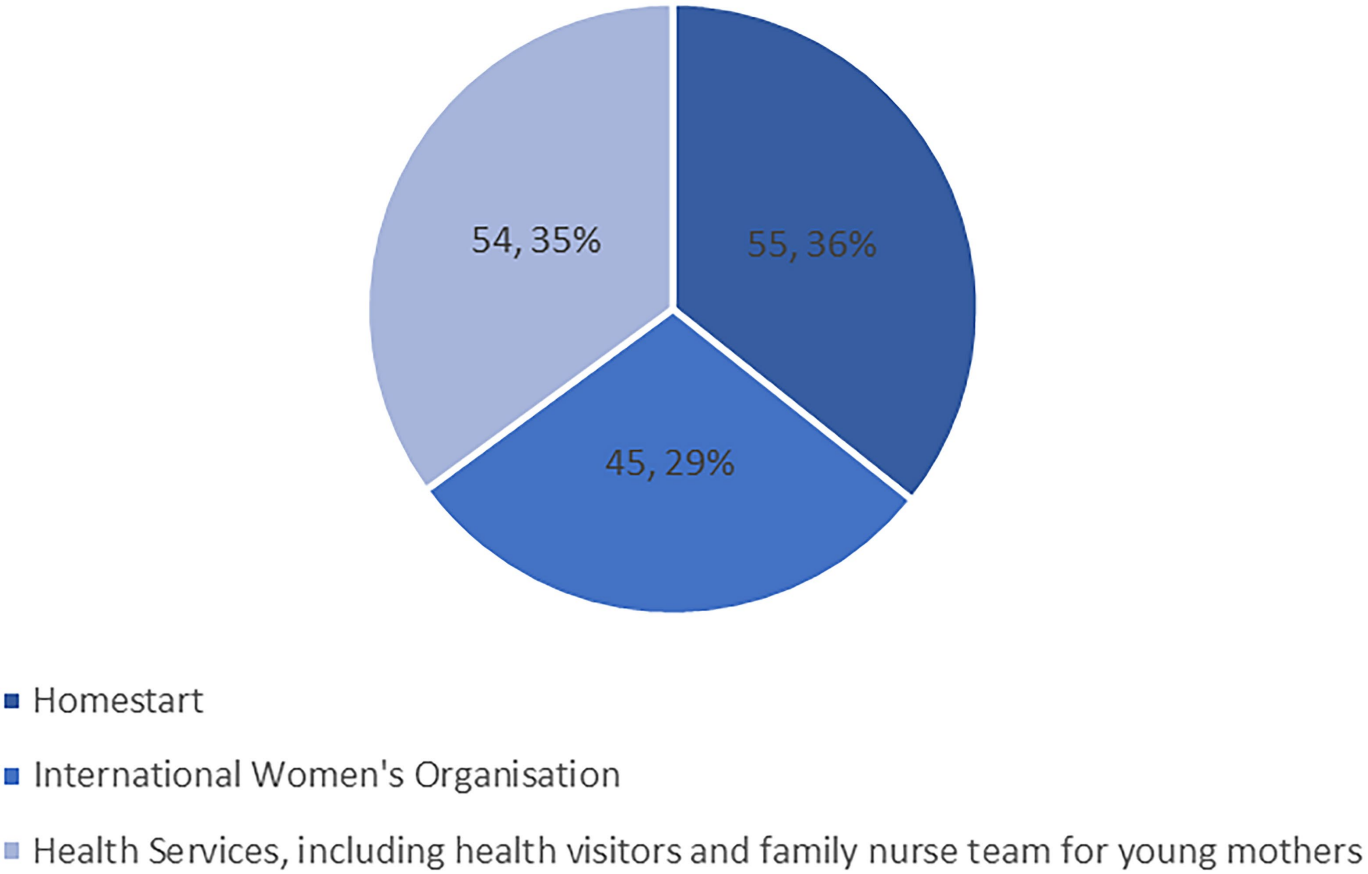
Distribution of Art Box referrals (giving number of referrals from each route and percentage of total).

**Figure 2:**
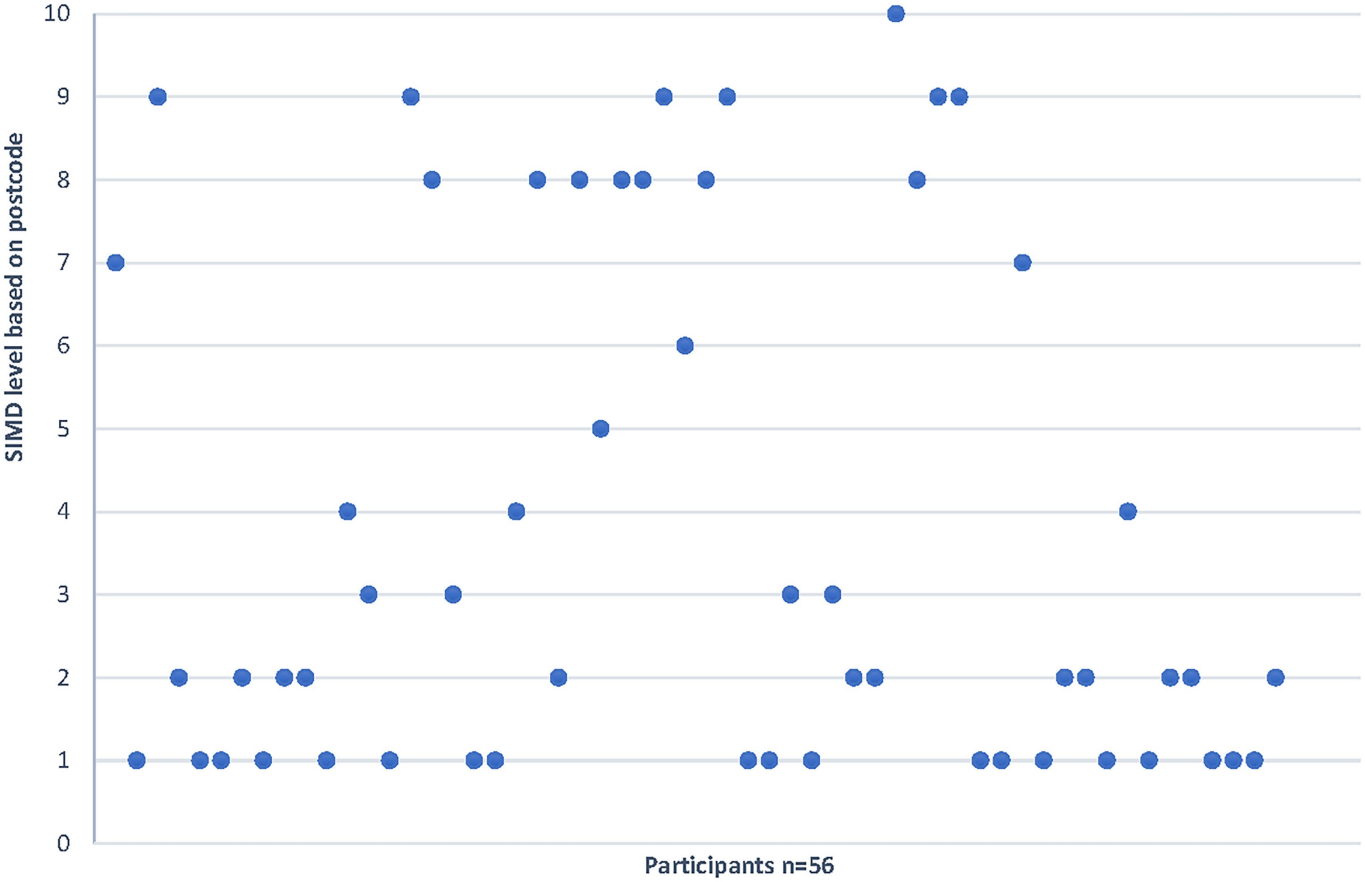
Scottish Index of Multiple Deprivations (SIMD) level for participant referrals (where 1 is the most deprived decile, and 10 the least deprived decile).

In total, we delivered 154 boxes to families during this stage of the project throughout the summer of 2020. Figure 1 shows the distribution of referrals among the different routes. Where we held address information for participants (some boxes were distributed directly by the charity partner) we were able to look at the SIMD index (SIMD, 2020). This showed that 57% of the families were in the first or second decile, that is in the most deprived 20% of the population (Figure 2). This suggests that the art boxes may be useful in reaching families facing inequalities. However, there were also referrals within areas of least deprivation, reflecting the pervasive risk of perinatal mental health difficulties, regardless of social context. Only one referral was for a father and the rest were all mothers (although the thematic analysis highlighted that some fathers did become involved with the art making at home).

### Intervention

**Figure 3:**
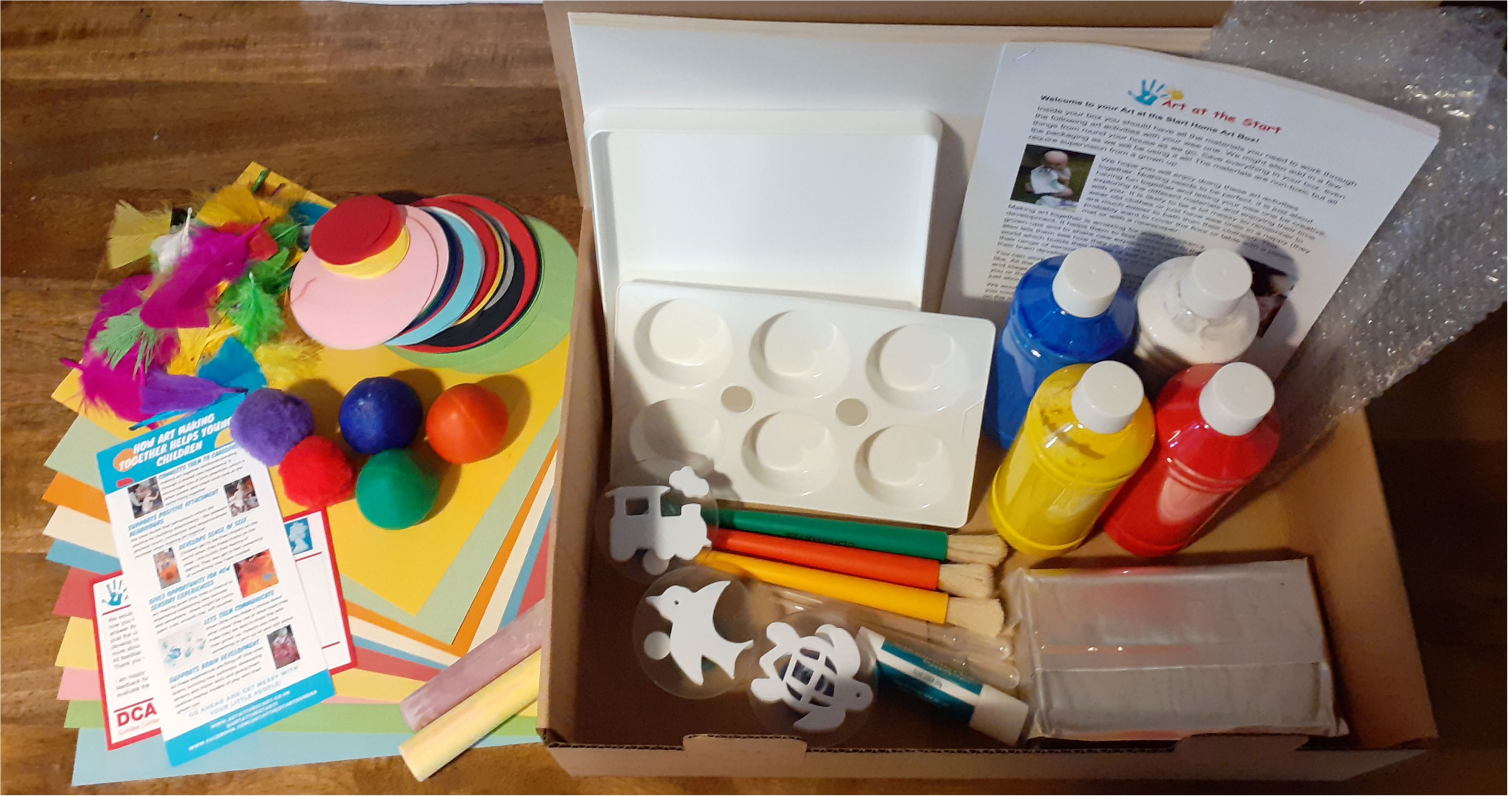
Contents of an Art Box.

**Figure 4:**
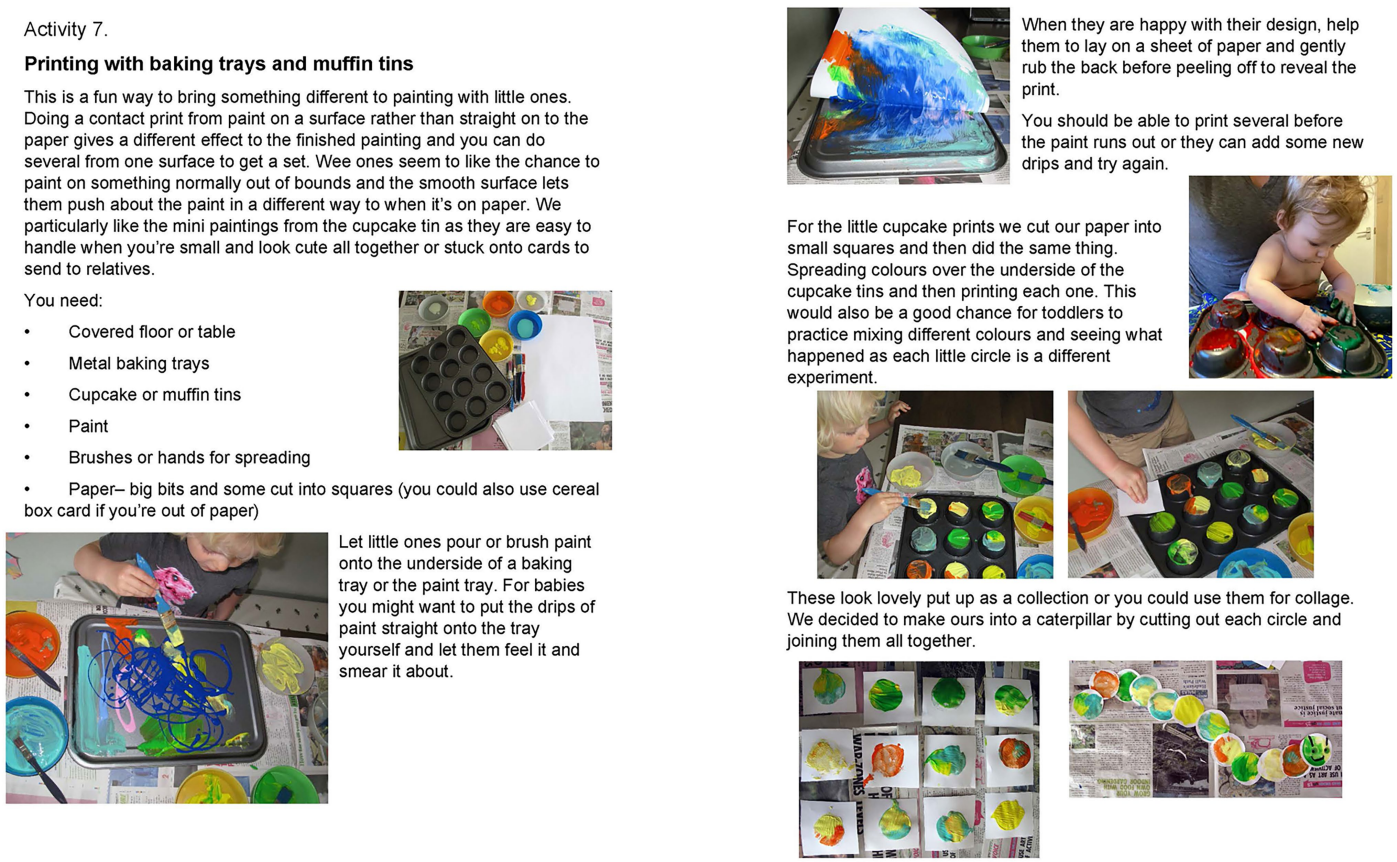
Sample pages of Art Box worksheets.

The art boxes were designed by the team’s art therapist with the aim of encouraging parents to make art together with their infants, fostering connection through playful and creative shared experiences. Although the art boxes are not themselves art therapy, as they are completed outside of any therapeutic relationship, they took inspiration from our research into the benefits of joint art making during art therapy sessions. There were a broad range of baby-safe resources (Figure 3), including paints, brushes, stamps, clay and papers. Alongside was a booklet with information of why art making together is beneficial from a psychological perspective, that could be considered psychoeducation, guidance on the practicalities of getting started with very young children, such as ways to set up the space, and then 12 guided activities with lots of flexibility for age and stage (Figure 4). These were documented with photographs as well as written instructions in case literacy was an issue. All activities focused on doing the art together. Although not in person, we still consider the art boxes to be an intervention as they were supporting families where a need had been identified and sought to create change, by encouraging participation and guiding them through the art activities. There were clear instructions and well as direction on ways to engage. For example, parents were encouraged to follow their infant’s lead and respond, or to verbally describe what infants were choosing. The box also contained a written participant information leaflet explaining the opportunity to take part in the wider research project and giving the researchers contact details.

### Data Collection

Our data collection included a variety of different methods. For reporting to our wider organisations and funders we had included anonymous postcard feedback, with a simple survey and space for open feedback (and a tick box for consent for the feedback to be used for research), in every box. These results are included in Supplementary Material and, although positive, are limited in number (*N* = 57), and by the fixed scope of questioning. The main data gathering for this research is based upon in-depth interviews with a subset of participants and referrers, alongside gathering informal feedback to include wider perspectives. Interviewees were recruited during referral, where parents were asked to tick a box if they were willing to be followed up for interview after they had used the boxes. This aimed to minimise responses being biased by whether parents felt positively about the art boxes, although they would still have been able to opt out of being interviewed by not responding to our calls. We are also aware that those agreeing to interview, even in advance, may also be those more inclined to be favourable to the intervention. Given the specific population focus of our topic we estimated that between 15 and 20 interviews would give sufficient breadth of subjects and this was confirmed by the richness of the data (Braun and Clarke, 2021, p. 34). Twenty parents agreed to interview on their referral form and we interviewed 16 parents, based on those who we were first able to contact by phone. The interviewees were all mothers with infants between ages 0 and 3. There was an even spread of participants among the referral organisations, meaning that there was likely to be a balance between families facing isolation, families facing difficult social circumstances and families where there were mental health or attachment difficulties. However, our consent process was based on interviews being anonymised and not linked to referral information, as our small geographical area would risk interviewees being identifiable, so we are not able to compare the interviews with demographic data. In terms of previous experience, 14 of the parents had never tried making art with their baby before, and two had tried to a limited extent or with older children. When asked if they had visited the arts venue in which Art at the Start is based (Dundee Contemporary Arts) with their baby, none of the parents had, although one had been previously with older children and two had been for social visits to the café bar.

A further level of feedback data was based on interviews with referrers from across the range of roles in our referring partners. We reasoned that it was important to hear both directly from parents and from informed observers of their relationship, to best capture any change in parent-infant engagement. Including referrers helps give an indication of whether the feedback we got from our interviewees was reflective of the wider group experience. Referrers had knowledge of the general picture for the families they work with and had seen the boxes used by a number of their clients. We interviewed four referrers—an NHS health visitor, an early years support worker, a community group support worker and a family nurse. The referrers interviewed also stated that the families whom they had referred had not made art together before. This brings the total number of interviewees to *N* = 20.

A semi-structured interview method was chosen—with an interview guide of potential topic areas (see Supplementary Material for the interview topic guide) but no set interview schedule. This allowed the researcher flexibility to respond, follow up or expand on comments (Rubin and Rubin, 1995) and to clarify questions or answers (particularly important as English was not the first language of all interviewees). For all participants, the interview began by discussing the information provided in the participant information leaflet, giving the opportunity to ask questions, and obtaining a recording of verbal consent.

Alongside the transcripts of the interviews, we have included informal feedback from both parents and referrers, gathered throughout the project in the form of emails, text messages and social media messages (43 records). The participant information leaflet in our boxes gave our contact details and information on how any feedback (formal or informal) would be used. Where feedback was passed on by referrers, we made sure to clarify that parents were happy for it to be included in the research. Parents also shared photographs with us through email and on social media. Researchers have successfully used images in social research, for example by producing and analysing text-based descriptions of participants drawings (Savoie-Zajc, 2005) or by having participants take photos that form a basis for discussion (Radley et al., 2005). Images may be used to develop a shared communication between researcher and participant (Harper, 2002) and there is increasing interest in using visual methods to allow participants to communicate their perspective (Gauntlett and Holzwarth, 2006). In our case these images were ‘naturally occurring’ rather than elicited, but as these images had been chosen by parents to share, it was our understanding that they would therefor reflect something of importance to parents about taking part in the project. With the parents’ consent, these images (112 records) were included in the analysis alongside the text data.

Qualitative analysis was undertaken using Nvivo software (version 12, 2018) which allowed us to include the images alongside the transcripts and screenshots of informal feedback. We used thematic analysis to reach a meaningful understanding of the data within this rich data set and took a reflexive approach as outlined by Braun and Clarke (2006). We took an inductive orientation to the coding, starting from the data, but with awareness of our own theoretical understanding of interactions within early relationships. We coded for both manifest meanings (what the parents were directly telling us) and latent meanings (in our case where examples of behaviour they described seemed to evidence psychological change in parent or child as defined by our theoretical underpinning; Braun and Clarke, 2022). A preliminary analysis of 10 parent interviews (Armstrong and Ross, 2021) had identified initial themes so we had these in mind, but we coded the entirety of data again and introduced new codes as needed. The full data set was coded by the first author and then these codes were reviewed by the second author before they were refined further. Following this, codes were organised into themes that made meaningful sense of the entirety of data. These will be discussed in detail here and examples given.

## Results of Thematic Analysis

**Figure 5:**
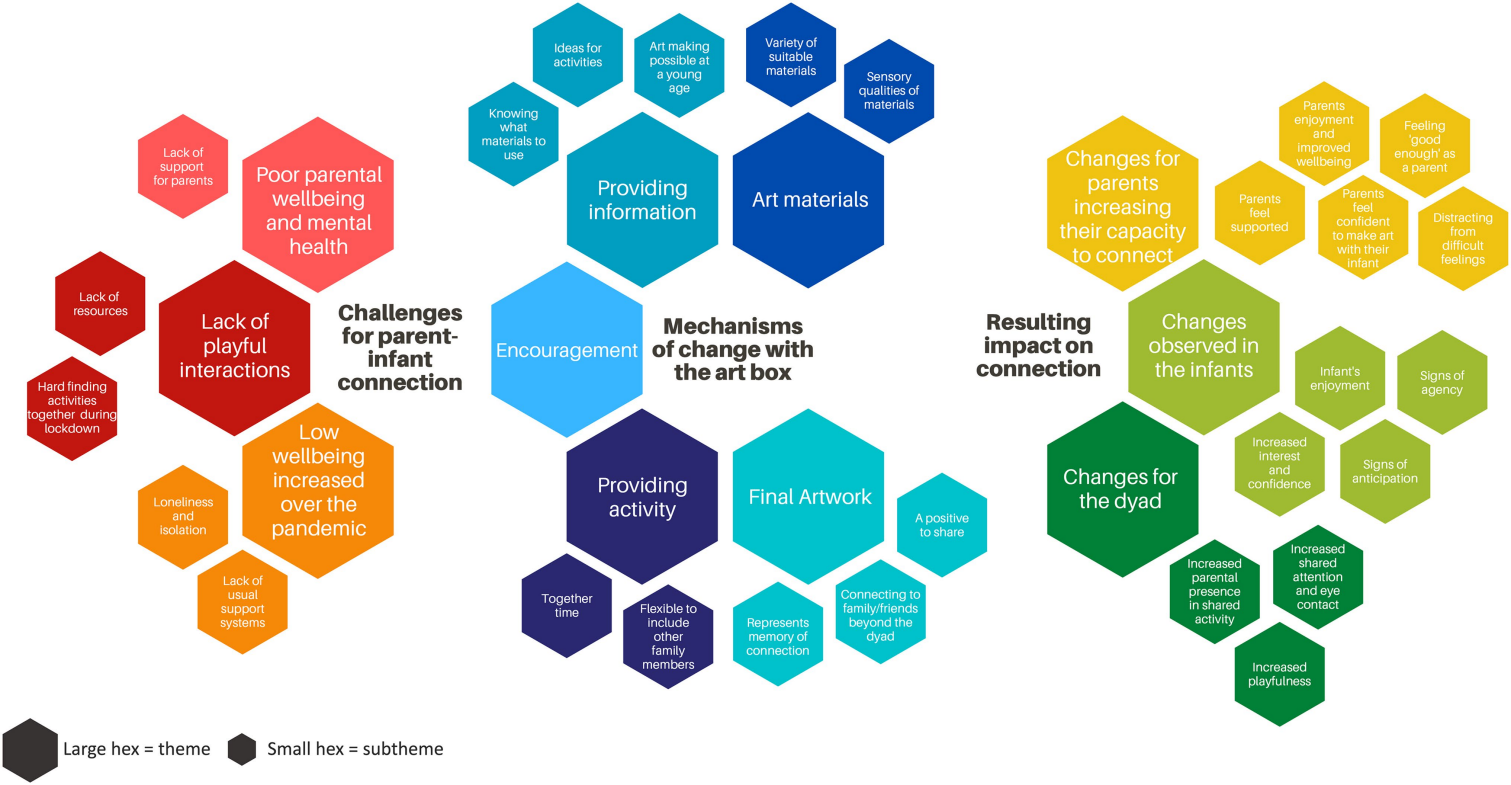
Diagram describing organisation of themes from thematic analysis.

The themes we have developed from coding of interview transcripts, informal feedback and images are wide ranging. They reflect the depth with which the participants shared their experiences as parents (including of their time over lockdown), how they had used the boxes, and how they felt about the art-making process. We have found it useful to organise the themes into groupings under three larger overarching areas that look at the **challenges for parent-infant connections (1)** both during parenting generally and in lockdown specifically, the **mechanisms of change with the art box (2)** intervention, including practical and psychological aspects, and lastly the **resulting impact on connection (3)** as reflected in the changes seen for parents, infants and the dyad. This structure allows us to make meaningful sense of the entirety of the data while keeping the parent-infant relationship as a central focus and offering clarity about what specific mechanisms within the art making may help to create change for dyads. Figure 5 outlines this structuring of themes and subthemes. These are described more fully below with a selection of quotes that are representative of the larger body of data (Figure 5; For ease of reference, as specific themes and subthemes are mentioned in the text they are given in bold as **theme**, and **subtheme**).

### Challenges for Parent–Infant Connection

This category gathers together themes reflecting difficulties in the families’ lives that we would recognise as having a large impact upon the parent-infant relationship. This gives us a picture of some of the areas of need which we hoped to address with the art boxes. Parents and referrers talked about **poor wellbeing and mental health** that was already an issue outside of the pandemic.

*She had really low mood and anxiety - was anxious before covid (referrer).*

We know that these mental health difficulties can limit a parent’s availability to form connections with their infant. An important subtheme emerging from this area was parents’ perception that there was a **lack of support** in their area.

*I don't even know if there would have been anything that I could have gone to anyway. Like I don't ever think there's very much around for mums. Like not much stuff to help them (parent).*

An important theme coming from both parents and referrers was that **low wellbeing increased over the pandemic**.

*I think we’ve seen Covid has had huge impacts on our clients. I would say increased levels of anxiety, isolation, loneliness. And you know, anybody with diagnosed mental health issues have deteriorated (referrer).*

One referrer also brought up the potential for increased stresses for the infants over this period and gave examples of infants now facing heightened separation anxiety or lacking in socialisation.

Subthemes here added detail to some specific issues for parental wellbeing. Parents described **loneliness and isolation**, and particularly those alone at home with an infant, found it extremely challenging during lockdown not to have other people with young children to interact with.

*It's been like completely shit. Like we’re on our own just. There's nobody here but me with the baby. I hadn't really met anybody before it started, so to actually have someone, like to meet or that with kids… So there's not any really friends to see or anything so. It’s like just not really been good (parent).*

For some parents this then also led to them feeling guilt for feeling lonely:

*it was like so lonely. I feel guilty because I'm not on my own, I'm with her, but, Yeah, just like long, long days. It’s a bit long with a baby anyway, but long and yeah, just feeling by myself, You know? (parent).*

One factor which may have contributed to this isolation was a sense of feeling different from their peer group when they were the only one with a baby. This may have been particularly relevant to the younger mothers in our sample. This added to a feeling that their experience of lockdown was very different from others.

*I’m the only one of my friends with a baby, and then I speak to them and it's like they have absolutely no idea how it would be like for us to be by ourselves. They're like not even finding it that bad and they’re like happy, maybe, that they're not, not having to go into the office. But then for me I'm like just us at home all of the time. It's like they can't even imagine that (parent).*

Adding to the experience of decreased wellbeing was the parents’ **lack of usual support systems**. The referrers described their own reduced capacity to support families over this time, particularly the lack of face-to-face meetings. But on top of reductions to services, families also experienced a loss of informal supports from family, friends and other parents. For some parents the lack of support which they perceived was a source of anger.

*No, nobodys did anything. I think it’s terrible that so many of the things that should be on for kids have just been totally stopped. You know, it's like, as if they just don’t think about how things are for us, nobody's thinking about kids, about…you know (parent).*

The last theme identified as a difficulty for connection was a **lack of playful interactions.** Some of the referrers expressed concerns about witnessing a lack of interaction and play in houses, and their difficulties in getting families to engage with suggestions. They described issues with televisions being on in the background and limiting interaction, or parents not knowing how to play.

*I came in this job thinking everybody knows how to play with their children but they don’t. And that is probably one of the biggest things that we all need to be supporting the families with (referrer).*

Parents focused more on the specific difficulties of play during lockdown, which was possibly easier for them to talk about, but some did express finding it hard to play more generally and linked this with mental health difficulties.

*if you're not feeling good, it's like, actually too much to try and think of something yourself to do (parent).*

The lockdown specific difficulties for play included parents’ observations that it was **hard finding activities together during lockdown**. They described long days, feeling bored, and struggling to keep themselves and their baby occupied.

*like you're on your own, I guess, at home and it's just really us so that it's hard to think of what stuff we might do together. It feels quite long, I guess. A day like. The days are going, yeah, go really slowly and you don't do, like you’re playing with their toys and you're trying to do something fun for them. But actually, if you're not really able to go out and join in with stuff or meet your friends like there's just not that much, you know* (parent).

Parents with older children in the house highlighted the added difficulty of finding something to play with different ages at the same time and the need to divide their attention. The lack of parenting groups that might normally be on, such as library groups doing stories and rhymes or parenting groups, came up in most of the feedback. Some had tried virtual alternatives but not found these to be less useful.

*that's one of the main problems, I think, was that all the stuff that would normally be on for babies, all of that just stopped. And I know like it's nobody's fault. And then sometime some were, were trying to do stuff, like over the computer with like Skype and stuff, but, I don't really want to be doing computer stuff. I went a couple of times to, em, like a mum and baby group that we were thinking about before, and joined in with their, I think it was on like a Facebook thing, not on Skype… so I tried that, but it's just, It's no… I mean, there's no point in it for [baby]. And then just on a screen, just looking at other mums, you know (parent).*

A **lack of resources** was also an issue, with parents describing material issues getting in the way of play with their infants. There was limited availability of toys or art materials in the house, sometimes due to not knowing what resources would be suitable or having limited access to shops during lockdown, but mainly due to their financial difficulties. A high proportion of the families were on low incomes and financial difficulties may have increased over the pandemic with some reporting partners losing work. A resource issue existed around physical space leaving families feeling restricted in how much they are able to play in their homes, particularly for those with more than one child. Another issue was around internet access, with one referrer giving an example of families having internet cut off due to their financial circumstances. This would result in difficulty for families in accessing some of the ideas and help for play that were put online.

*our house is small, so there there’s not that much space for us to have room in the house and then…We have toys, but not so many. And hard for them to share those (parent).*

### Mechanisms of Change With the Art Box

Within this category we have organised all the themes where parents and referrers described specific qualities of the Art Box intervention which we would consider to be mechanisms of change. These help us to think about what the process of using the box does and why. An important theme here, brought up by all participants, was **encouragement**. Parents described how it gave them a push to try something that they might not have otherwise, or an extra bit of motivation. Some described trying it because they felt they ought to and then being surprised when they enjoyed it. There was something useful about having everything they would need ready to go and a specific thing to try for overcoming barriers to having the first attempt.

*I always felt that I want to be doing things, but just finding that I didn't really have like the energy or I wasn't really enthusiastic to do them. So sometimes you just have to start the thing. And then it will be good. And so having that idea and this nice box of stuff, that, that gave me that (parent).*

Every participant described how they had tried various activities within the box and referrers also described families giving it a try where they may not have expected it.

*And some of the families that I thought they would not use it, they did! “Oh yeah, we have used it, we have done finger painting” and they were enthusiastic about it. They were quite impressed with it. So it was all really good feedback actually from the families (referrer).*

**Figure 6:**
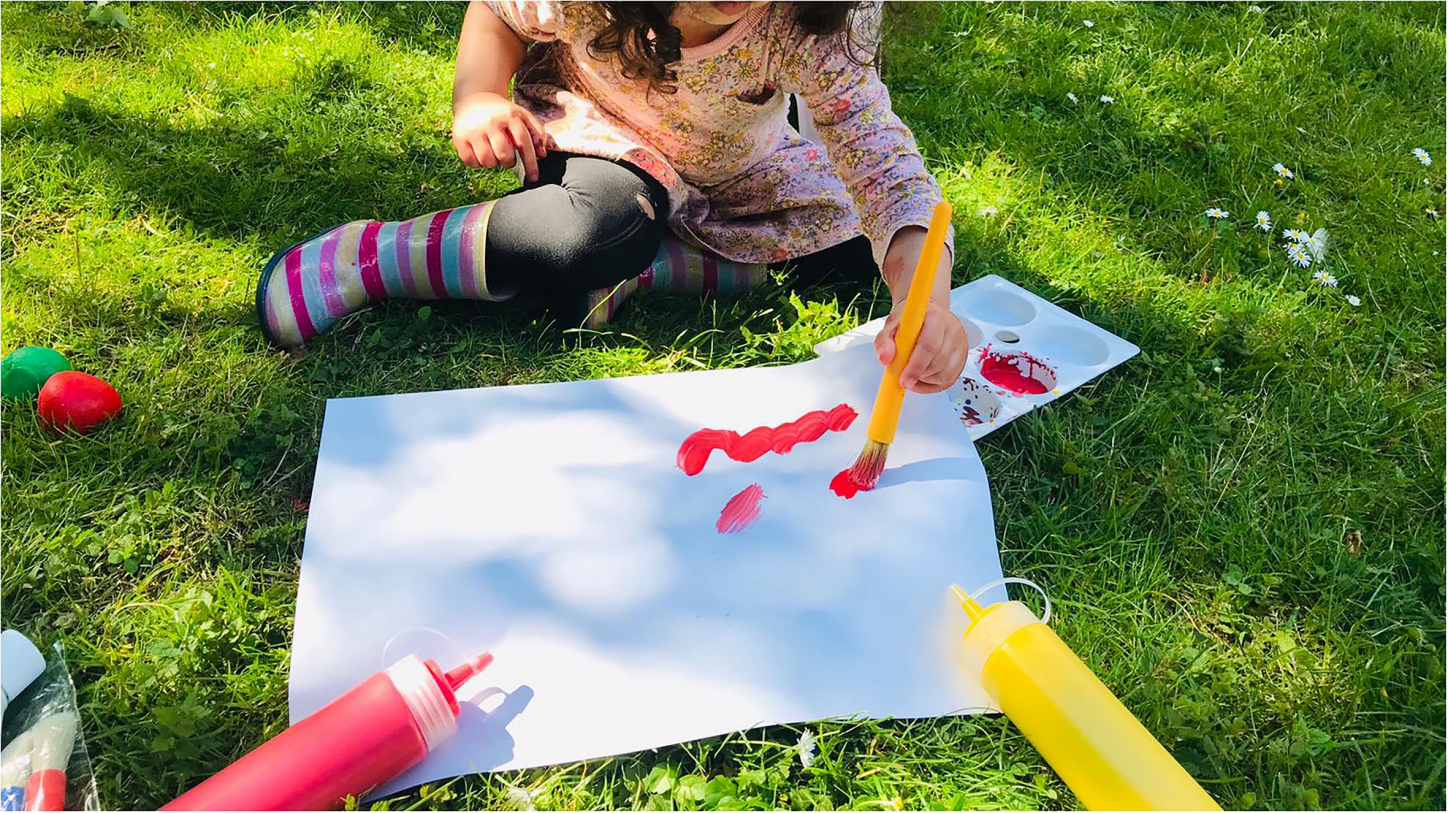
Image shared by parent of infant trying art making (image cropped).

One referrer was able to give very specific examples of a couple of families where she could clearly see the resource had been used. One mum had surprised her by getting out all the materials to do footprints and the baby obviously having experienced it before as they were joining in and painting their own feet. She observed another toddler making shapes using the crayons and felt she looked familiar with the activity, indicating the family had been engaged with art making for some time. This sense of encouragement to try the art making is also reflected in the images which we were sent back where a large number of these we classified as showing the infants trying the art materials (e.g. Figure 6). This perhaps reflected parents desire to share that they had tried the art box.

The art boxes were **providing activity** and parents described their appreciation at having things to do in their day together and some activity that was planned for them—*something to keep us busy and make us feel better*. This theme connected to some of their frustrations over lockdown with boredom and lack of activity.

*I know it's been really nice because, I don't know, just having a focus, like a thing that we're both meant to be doing, gives you that something busy in your day. Um. Gives us like, I guess time we're not watching telly we're just like, doing something and I think there's not been enough stuff in our day that we've been doing (parent).*

Parents appreciated that it was possible for the activity to be **flexible to include other family members**. Parents told us about involving the other parent, their own parents or taking the boxes to friend’s houses when restrictions allowed this.

*These boxes are not just art boxes, these are boxes that bring families together, because me and my husband have been doing these activities with the children, and guess what Gran joins in too (referrer).*

In particular, parents spoke of being able to include older siblings in the activity too and described how managing different ages had been a challenge, so this was a useful quality.

*I think it was nice. It was like easier to do this with them both than I think it is to do other stuff…maybe because it was new things for both of them. Actually, they got along a bit better doing it and it was easier for me (parent).*

**Figure 7:**
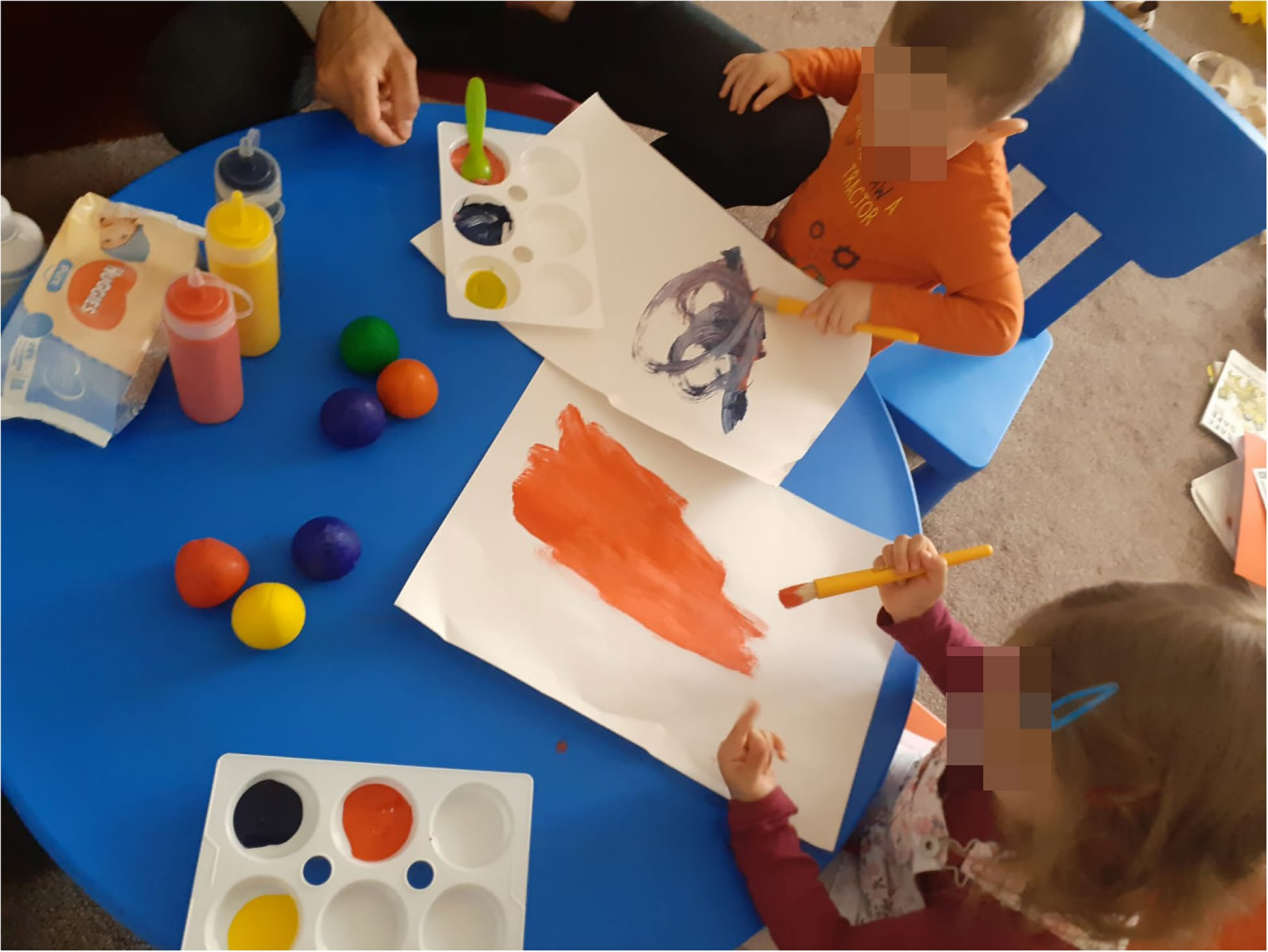
Siblings using the art box together (pixelated).

Several parents described less antagonism between siblings while doing the art together. This theme also included images sent in my parents of siblings making art works together (example Figure 7).

A subtheme about having activity to do together was that this was an activity about **together time**. Parents described how these activities were ones which they were meant to be doing together with their child and that this was something both different from normal and which they valued.

*I think the art it did make a difference, but it's like a thing that, like I can't think how to say it. Like it, ah, like it brings you into something so you both are doing? Or something that, like the point of it is to do it together, you know? (parent).*

This was also reflected in comments from referrers who had noticed that families considered the art making time to be special time for the two of them.

*It’s been interesting actually one of the parents I spoke to just this week said in one of her observations, she was like ‘it's really quiet when we're doing it’. And I said Oh what do you mean, like they're not talking. And she said ‘no because normally I'd have that tele on but for this I turned it off (referrer).*

**Figure 8:**
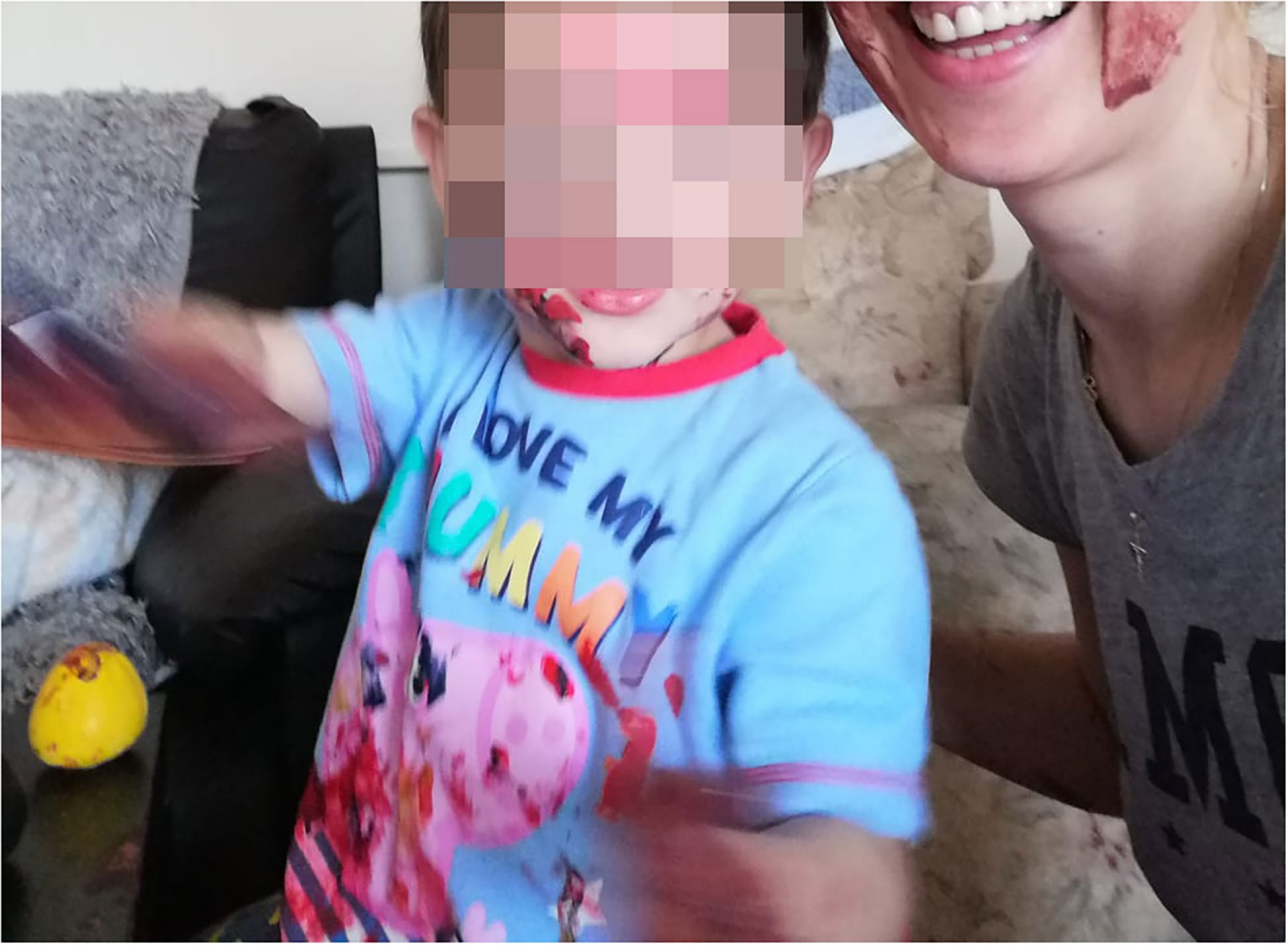
Image shared of parent and infant doing activity together (image cropped and pixelated).

This sense was also captured in some images returned which showed the mother and infant together during or after art making (e.g. Figure 8). Both parents and referrers spoke about parents setting aside specific time in which they would do the activity together. This showed the value placed on this together time, and also that it helped them to have some structure within lockdown.

*she really recognised herself that she was missing routine. So she had the two days, so she would spend the morning with him doing arts and crafts and things, then and then afterwards they had good fun to put him in the bath to get clean again. So yes, she really found it beneficial (referrer).*

The third theme relating to mechanisms of the art box intervention is the physical provision of the **art materials** and the different qualities they have. Many parents and referrers appreciated being provided with the resources which they may not have been able to afford or find otherwise. Some parents commented that they liked having the materials chosen for them and particularly that there was everything they needed for the suggested activities, so they did not have to add anything in themselves. Others commented that there was plenty provided, so they had been able to keep using them after finishing the suggested activities. They described the different materials that they and their infants had particularly liked and gave examples where the physical qualities of the materials were particularly suitable for babies.

*She seemed to really like the big crayons that you can, she can hold in their hands now it's nice and like she could just hold it (parent).*

**Figure 9:**
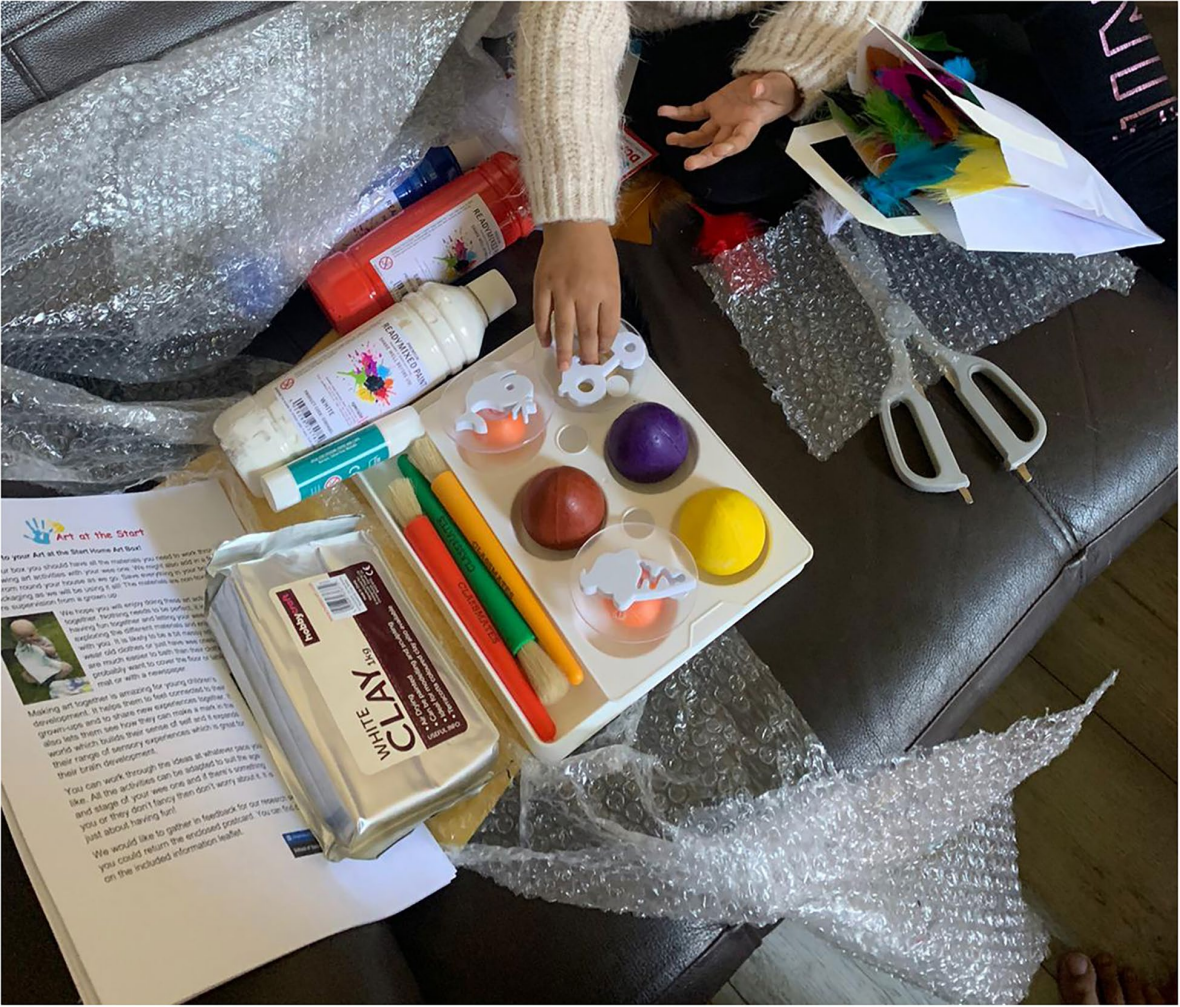
The art materials laid out (cropped).

This appreciation of the materials was visible within the images, where we got a high number sent in which showed the different materials (e.g. Figure 9). These kinds of images were unexpected to us but may illustrate the importance of the materials to the families.

Many parents brought up was about **variety of materials** and enjoying the mixture that was on offer.

*With toys it’s a bit repetitive…it’s always the same games that they choose right? With the art stuff it can change so much to different, you know, we could be doing hand prints or we could be doing clay or we could be drawing a house, or whatever, so you know, it changes so much, and oh the feathers, you know, we had so much fun (parent).*

One parent described how some of the materials like feathers their baby was able to explore now but would be able to use more when they were older so the box would adapt with them. When describing favourites, it was interesting that several parents observed that their babies and their own preferences were different but that this was a positive thing.

*I mean any of the ones that were covered in, like slimy paint for him, I think. But for me, probably the ones that gave me something nice to keep. So probably our favourites were like the opposite and that he liked something messy and I liked something like the clay handprints. That I will like, always keep those. But I think, probably that's quite a good balance, isn't it? That there was things for both of us in there? (parent).*

This idea of being able to meet a variety of needs through provision of materials with different qualities was also reflected by parents with siblings of different ages.

*I think he, it's hard to tell with the baby what was their favourite, but I think just like maybe more the feel of everything rather than the, like rather than the actual particular activities. Like the colours and the feeling you know. Whereas for my older one he’d see the idea of what you'd made and try to copy it (parent).*

Parents commented on the **sensory qualities of materials** and noticing that their infants were liking their tactile nature.

*I think she just liked painting and the chance to get your hands in and like, she liked the feeling of it, and I notice by the end more confident to like, to put it onto the paper and be like moving it about with their hands. I think she just really like that feeling (parent).*

**Figure 10:**
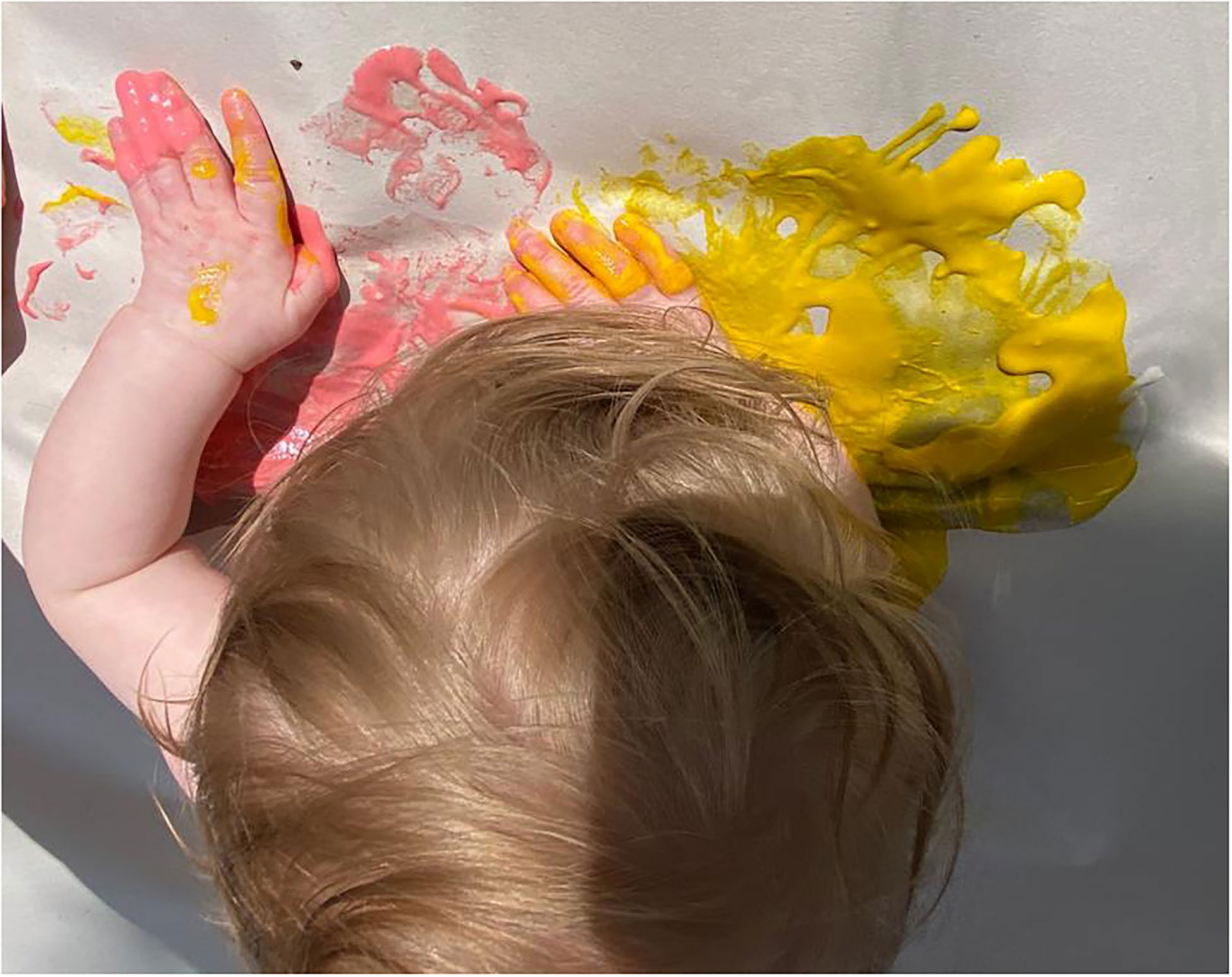
An image of messy paint exploration.

Within this theme, mess was something that came up a lot and was also reflected in the number of images we received showing the messy materials or babies covered in paint (see Figure 10).

Some parents commented about some initial nervousness about the mess while others spoke about the mess very positively as being fun, or expressed pride that they had been willing to try it.

*I think like one thing I've notice that's different is I'm kind of prepared to get quite messy, so I'll put on clothes I really don't mind getting covered in paint and so actually, even when she's like covered in paint or something, I don't mind if she wants to come and cuddle me (parent).*

Parents described ways the art box had helped by **providing information**. Within this were comments about getting practical knowledge as well as art ideas and for some parents more understanding about how to be with infants when making art together.

*the idea was there because, you talked a lot about it being, about the, like, about the doing it, so I felt a bit more relaxed (parent).*

Parents all said that having the **ideas for activities** contained in the booklet had been important for them, some saying they had been a bit nervous, or would not have been able to give it a go without these ideas and others just how it had helped them by giving them new ideas. Several parents described that it helped to have an ideas for a starting point even if they then took the activity in a different direction.

*I think it helped having just ideas 'cause it's hard sometimes to just think of ‘What would the baby do?’ like ‘How would your baby do this?’ I think if I picture making art with kids and I’m thinking of like drawing wee stick people or something and actually, that's not what the babies are doing. So it was good to see those ideas of things which were more about the messy like fun side of it rather than drawing. I liked things I wouldn't have thought to do like the one where you were on like the cupcake cases underneath and printing on and then like doing it onto the bubbles, so I guess it's all kind of just paint but using something just to make it a bit different each time. It was nice having those ideas (parent).*

As well as ideas for specific activities, parents talked about getting information for **knowing what materials to use** with infants. Not knowing what would be suitable was something that had prevented some of them from trying art and several expressed being reassured by the information about what to use and how to use them safely.

*giving me all of the stuff so that I definitely knew that those ideas were safe and that like they would work and everything. And it told you how to set up and stuff (parent).*

Another way the boxes gave information was by letting parents know that **art making is possible at a young age.** Parents described how they had not thought they were able to do art with a baby and that getting the art box had let them know that this was possible.

*When they’re wee, I don't really, like I didn’t really know what you would do together in art like. I know that they can't draw, so I don't know, I wouldn't have known what stuff that we could do really and what was the right thing for that age? (parent).*

The last theme is the **final art work** itself. Art making may be unique as a form of play in having the potential for a final physical end product and this was something the parents seemed to value.

*I liked that 'cause you got the end print to keep and aye it was nice to have, like to have that, to have something that felt like a thing that we'd keep (parent).*

**Figure 11:**
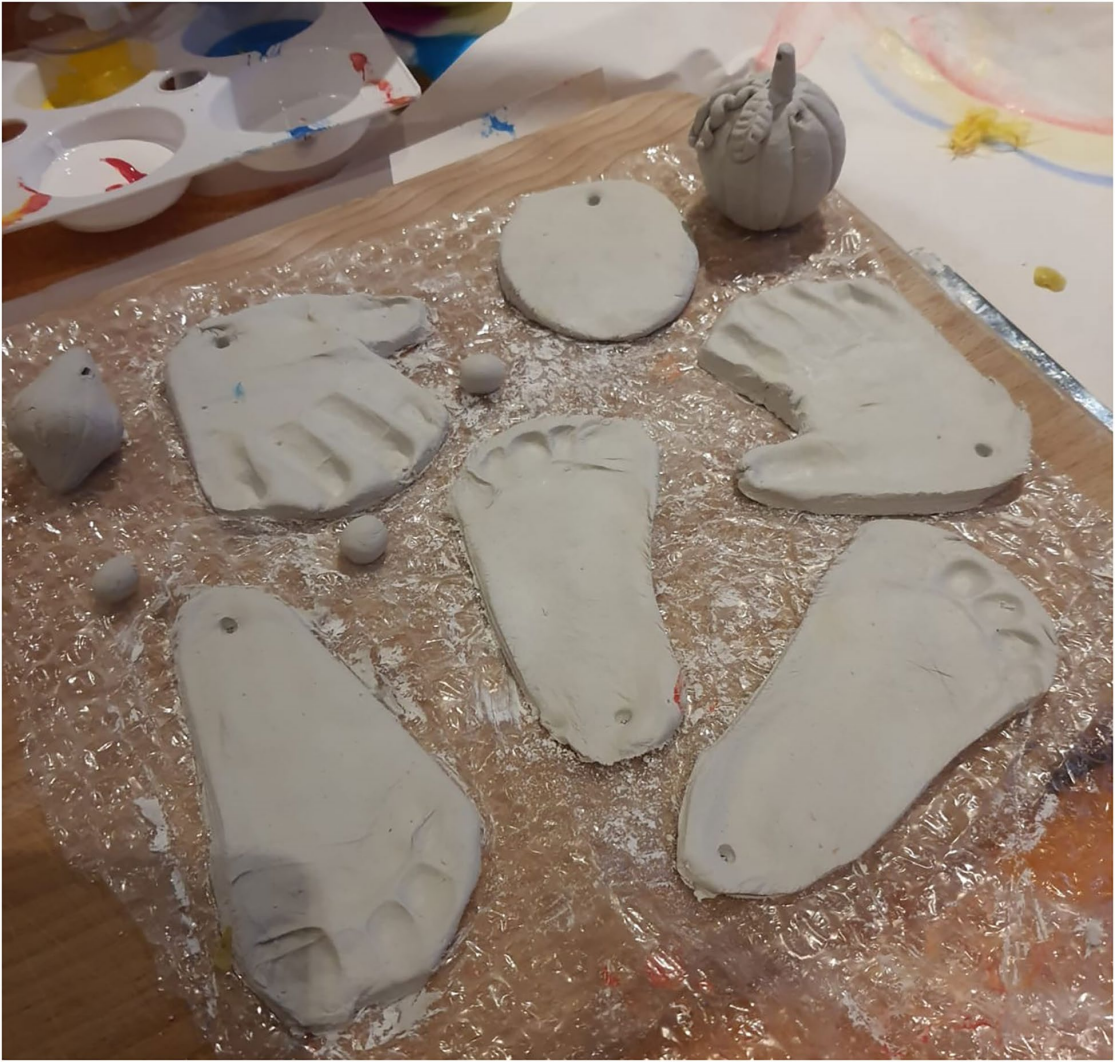
Final art work, hand and foot prints.

The large number of images showing final art works also connected to this theme (e.g. Figure 11), including some images where parents had displayed the work in their home.

Some parents described how the art works were a nice memory of doing something together—which we felt was the way a final art-work **represents memory of connection** and this is able to extend the experience beyond the session.

*what I notice is I can then look back on stuff that we've done together. So like you know, a few weeks before, and that thing is still there and it's a nice, It's a nice thing to have and like to put up (parent).*

This may also be a memory for the infant, with one parent describing putting their art work up for their baby to see.

*I’ve managed to put a couple up and there’s one, like in a bit where it’s like her space, there’s something that she’s done. I do not know if they recognize it, but certainly something colourful that’s theirs (parent).*

By having a final product which can be given to others or shared as a photograph, parents described the art works help **connecting to family/friends beyond the dyad.** Many parents were giving pictures as gifts to friends and family, especially grandparents. There were also a lot of comments about sending photos or sharing these on social media.

*I think people even when like when I send people photos of us doing, um me, my baby, doing art together. Everyone that I sent a photo to all said please can you send me some and they really appreciated getting it and it was nice to make it and it was kind of a talking point that wasn't just about, oh look, she's able to, you know, I don't know, she was laughing today or something’. Like it was something else to talk about that was about the baby and sort of involved other people* *(parent).*

This aspect seemed particularly important given that lockdown restrictions had limited contact with family outside their own home. A subtheme which extended this idea was about how the final images gave parents something **positive to share.** Some parents talked about how little they were doing during lockdown so this gave them something interesting to share and for others it was about having a positive where their family might be concerned about them or they might usually be sharing negative aspects of parenting.

*to share something nice that was going on. I think sometimes, if I was speaking to my mum on the phone and just probably all I was saying was like how bad everything was all the time and she's not seeing anything good, so this was like, the nice side of being a mum and the nice side of the stuff that we're doing and I was getting to share some of that with her. So I think she was relieved as well to see that it wasn't all bad that there were these nice bits (parent).*

### Resulting Impact on Connection

The final group of themes all capture changes following the intervention, which may impact upon connections between parent and infant. The theme of **changes for parents increasing their capacity to connect** was about parents describing changes in themselves which we see as signs that they would be more available for connection and that their capacity has increased. Parents described how receiving the art box helped them to **feel supported**. Additionally, the referrers also said that they appreciated having something they could offer to support parents when they were restricted in their contact.

*made me feel like I was able to do something to help the families as well. Because we all felt a bit helpless at that point. And we couldn’t go out. We usually have a lot of information to give to families so I was actually quite nice to say “would you like an art box?”. It was a nice thing to be able to do. It was nice to feel like we were helping them (referrer).*

A lot of our informal feedback was messages from parents with general expressions of appreciation that reflected a feeling of support through the boxes.

*We really appreciate your care and love for our kids (parent)*

One way in which this feeling of support manifested in the parents’ comments was in their descriptions of how they had felt thought about and in comparisons of the art boxes to a gift.

*We did that, we opened the box together, and both of my children and we open this together and look through what's there. We feel just blessed to receive this. We, we felt that we were thinking of (parent).*

**Figure 12:**
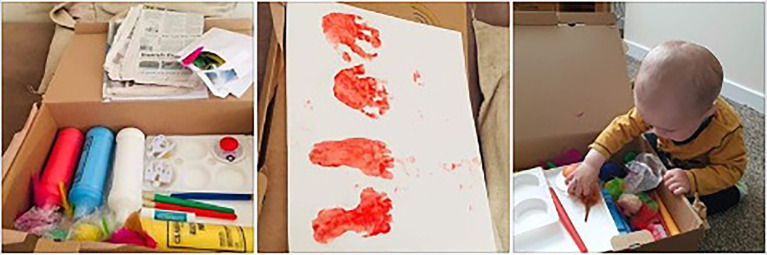
Image shared through social media of infant opening box.

Images we received also connected to this theme, where the image was of the parents and infants receiving the boxes as opposed to using them, for example, images of a dyad together holding the box. We also had several photos where infants were pictured in the process of opening the boxes (Figure 12).

**Parents enjoyment and improved wellbeing** was something described by all participants and that would make them more available for connection. This included more general increases in wellbeing and mental health from enjoyment of the activity

*I feel better doing it as well. Like I'm also having more fun. Not so bored. I'm just enjoying it. It's a nice thing to do together (parent).*

as well as specific improvements to issues around lockdown such as feeling less lonely or feeling more positive about their situation.

*it did actually make me feel just a bit better that everything, not everything is all doom and gloom, you know? (parent).*

Parents mentioned lowering their stress and feeling calm or more relaxed, including some specific comparisons to therapy.

*you’re feeling more like more like relaxed or that because I'm not trying hard to try and find something. It’s like something that's easy to play with her, you know, like so feeling like not like stress (parent).*

A connected subtheme captured **feeling good enough as a parent**. Parents had described some guilty feelings about their parenting and several parents described the boxes as helping them to feel like they were offering enough or doing the right thing, what we might think of as ‘good enough’ mothering.

*I think I feel more positive. I feel that I'm doing a good thing for my children. Where I felt while we were at home with no things to do that maybe I'm not doing a good job for them and that they are not getting all the things that they need to do, to try. So for doing this I feel that I have done something for them and that makes me feel good (parent).*

Another way in which wellbeing was increased was by **distracting from difficult feelings.** By having an activity to keep busy with parents described that this took them out of their worries

*Like if the day is a bit stressful or something I guess it takes you out of your head a bit, doesn't it (parent).*

We heard that **parents feel confident to make art with their infant** following the intervention.

*I feel a bit more confident now I've had some ideas about, like ideas to copy. I know that he can do it, so I feel a bit more brave, to like, look up an idea on the Internet. And you see sometimes on Pinterest there's like ideas of things you can do with their wee feet and that kind of thing, and so, now we've got the paint and I feel that, like, I know that we’ll manage. It's nice to be able to do those (parent).*

This increased confidence was really evidenced by both parents and referrers who spoke about ways in which they had been able to adapt the activities or continue on trying new ideas.

*now I feel like I would be able to do more of that kind of stuff like make up ideas. Like we were already made up ideas of stuff. Really, he did the handprints and then I was making them into animals and that. So now I feel like I could make up more ideas of what we could do using it than before (parent).*

Every parent interviewed said that they had either already continued on making art together after working through the ideas or they intended to keep going when they finished. This will provide ongoing opportunity for connection once the intervention is finished.

*It's definitely changed and made me realize that I can do more with even at young baby. Then I thought, um and actually I enjoy it a bit more than I thought as well. Like I'm not really a sort of perhaps someone whose practical and kind of I'm creative, but I don't do things like that myself, but I would now much more. I think before getting the box, I probably wouldn't have done so much, particularly because I wasn't able to go to like baby groups where I might have been introduced to it. Yeah, so it's been really valuable for me. And I think, like my baby as well. I think it's really nice that she will now grow up like having done that sort of thing and hopefully we can keep doing it together (parent).*

The second theme covers the **changes observed in the infants**. The parents all commented on seeing their own **infant’s enjoyment** of the process, describing the playfulness they saw and infants appearing happy.

*I think like having more fun, you know? He was more…It is more like playful, and I’m happy seeing him get to do something. Like he's having a fun time. Do you get me? (parent).*

Parents also noticed that their infants were showing **increased interest and confidence**. Examples of this included them being more interested in feeling the different materials, or in making marks.

*Yeah she got braver, messier, the more we did it. Like to start with she was barely touching it, you know And then after a couple of times doing it she was just getting really brave and she was just sitting about in her nappy covered in paint (parent).*

One parent related this to her baby feeling more confident generally as well, and an improved experience when she was in nursery. A parent also observed that their babies concentration increased and one that their language had improved as she learned words related to the materials.

*I would think that maybe she got more confident to do it, and went to where it was, something that she would ask for. It's funny, I notice her, also like she probably started talking more while we were doing it. Like, we were trying to see like the names of colours and stuff and she was saying the names and, and, probably talking to me more and to her sister (parent).*

Parents described infants getting more excited or lively when they were getting the activities set up—what we would consider to be **signs of anticipation**.

*She would be like excited if she saw me getting out the box him like as if she knew 'cause I kept all this stuff in the box, using it as storage, and then when she would see me go to get that, I think she knew now what we were going to do and she was excited. That was really nice and yeah I guess like she, she seemed more interested in it and more interested in the different things (parent).*

These observations show that the infants have experienced the art boxes in a positive way and that they are looking forward to having another experience.

Parents also described **signs of agency** in their infants, by which we mean that infants were able to see cause and effect from their actions in the world around them, through making choices about materials, painting their own feet or deliberately trying to make marks. Art making seems to be a medium which has a lot of potential for infants to express and recognise their own agency.

*…you see them holding the brush more and trying, trying to do the paint trying, trying to make, to make this…sorry, trying to make the line with the paint (parent).*

There were also observations of the infants trying to get their parents to do tasks for them and follow their lead which shows they feel agency and ability to express this to another.

*…like always trying to get me to join in as well, so if, like I looked away and was on my phone or something he would turn around and give me something that I should be doing to do (parent).*

The final theme, and most directly reflecting changes in the quality of connected experiences, is about **changes for the dyad**. The first of these changes was an **increased playfulness** with parents reporting that they were having more fun together and being different to how they might normally behave during play.

*Maybe because I'm enjoying it too and she knows so she did, you know, like we're both like smiling and that. Like, you know, like being silly with it. Maybe… like, more like, funny, playful like that together (parent).*

This would reflect a positive change in the infants’ experiences with their parent that would enable more feeling of connection.

Parents reported their **increased parental presence in shared activity**. The art making was described as something to be done together and parents were spending time where this was their main focus. They described how sometimes play with toys was about keeping infants occupied while they do other tasks whereas this was something where they gave it all their attention and were active participants. They saw their infants responding to this increased involvement positively.

*I think of all their toys as their toys for them to play. Whereas for this art it was a thing for us all that we were meant to all do together. And that's different. Not something to keep them busy, maybe while I'm cooking. More, more sharing (parent).*

A number of parents also described turning off the television to do the activity so again giving more focus to their child.

*Yeah, like normally I guess I probably have the TV on, but because I wanted them to like concentrate on what we were doing, I turned that off. So it meant I really noticed when she was saying stuff (parent).*

Some also described the physical set up as being different as this was an activity where they were down on the floor with the baby and were needed to offer physical support. These different ways that parents increased their participation in play will have increased the potential for moments of connection.

Parents described specific examples of behaviours that they saw from their infant which are signs of **increased shared attention and eye contact**. These included examples where infant referred back to their parent, made eye contact, or initiated joint attention where both look at the same thing together.

*like he'll pick something up like a brush and then he likes to show the brush to me and then put it in the paint and then he looks at me, you know? (parent).*

There were also examples where parent and infant were engaged in the same task – what we would call shared goals.

*I would put my hands in as well to show her what to do and I know she likes it. She's like making a painting and then looking at me. And then I'm gonna showing her what to do and that so we’re like doing it both of us (parent).*

These are direct examples from parents of the specific moments where they are connecting as a dyad, that have been facilitated by the art making.

## Discussion

Our thematic analysis was able to capture some of the mechanisms for change within the art process. By giving materials, ideas, information and encouragement, the art boxes were able to overcome some of the barriers to positive interactions which parents described in their lives, drawing dyads into positive playful interactions. Some themes captured perceived mechanisms which we had been hoping for in the box design, such as providing activity to promote meaningful time together. Other themes we had not anticipated: for example, parents placed importance on being able to share with others beyond the dyad, either by including other family members in the art making, or in being able to share images of the art making with friends and family *via* smartphone. This may reflect something particular about this time during the pandemic (where older children also need attention, but conversely other family outside the home feel distant). However, sharing may have been an important aspect for these families anyway. Art works may be symbolic of a positive experience and by sharing the art works they are able to share this sense of themselves and of the dyadic relationship as positive. We might connect this to the theme of the ‘good enough’ parent and a desire to capture and share positive moments where they felt they were doing a good job, both for themselves and for their family members.

We were also surprised by some of the images returned. While we had expected to see images of some final art works, and pictures of infants trying out the art and looking adorably messy, we had not expected to receive images that documented all the different materials or the actual moment of receipt, and opening, of the boxes themselves. This shows us one way in which the images were able to draw attention to areas of the intervention which had been important to the families, through their choices of what to share. In these cases, we interpret the images of materials as capturing the high value participants placed on receiving the art box resources, and the barriers these families face in accessing such materials themselves. Having material art resources which are age appropriate and available in the home is important to facilitate this kind of play, so this highlights that any intervention aiming to encourage art making (i.e., *via* online content) without providing these materials is insufficient. The images of receiving the art boxes helped us to understand numerous participant comments which described the boxes as ‘gifts’ and ‘blessings’. In our interpretation, these both indicated different ways of expressing the value placed on being given the boxes, and how they helped parents to feel ‘thought about’ at a time when they perceived supports to be withdrawing, and some felt ‘forgotten’ as parents at home with young children.

In those themes focused on impact which describe changes for parent, infant and dyad, we are able to think about the ways in which art making may be well placed to maximise connections between parent and child. Without necessarily thinking in terms of their interactions, parents had made clear observations of moments of intersubjectivity between them and their child; those moments where their infant was able to express an interest and share it with them (Trevarthen and Aitken, 2001). What is positive is that parents reported responding to these connection-seeking opportunities, and also that they themselves experienced positive feelings from these interactions. This shows the potential within art making for interactional synchrony (Isabella and Belsky, 1991) where parents offer well timed responses and the dyad are having reciprocal, and mutually rewarding interactions. The evidence from the parents’ own perspective of feeling increased wellbeing may have allowed them to be more mentally present and available for this kind of interaction. These kinds of synchronous exchanges are fundamental in building strong attachment relationships (Svanberg, 1998). If art is able to facilitate these ways of relating, then we could potentially have a long-term impact beyond the intervention. The signs of anticipation from the infants shows they have come to expect a positive experience with their parent and the art box, and suggests positive associations to their parents, at least within this context.

It was interesting to see parents highlighting the changes that they saw in their infants. Some of these, such as infants showing agency, may seem to be more about the infant’s own psychological development. Indeed, they may indicate that the art making process is well placed to facilitate change in the infant’s capacity in these developmental areas. However, we would argue that parents noticing these changes, is itself a strong sign of potential for connection. All these changes reflect development of the infant’s sense of self (Rochat, 2003). When the parents are able to notice these behaviours they are recognising their infant as an individual with their own sense of self, with whom they then have the potential to connect on an intersubjective level (Stern, 1985). As art making is a new experience for the infants, with textures and colours to explore, it prompts clear reactions from infants, and may encourage parents to be curious about their infant’s experience. In this way art may be ideally placed to encourage parents to mentalise—where they are able to reflect about their infant’s mental states and intentions (Fonagy et al., 2004). This ‘mind mindedness’, where an infant’s behaviour is seen as meaningful, is shown to be associated with strong attachments (Meins et al., 2001). Working to increase parents reflective capacity can be successful intervention for attachment difficulties (Suchman et al., 2011) and is a focus within art therapy (Armstrong et al., 2019) so it is interesting to think art could have this potential outside of a therapeutic setting. We would suggest that this would benefit from further exploration.

We have looked at our results through our perspective of infant development but there are other perspectives which could also add insight, for example those from positive psychology which consider wellbeing. Fredrickson (2004) describes how positive emotions may ‘broaden’ an individual’s repertoire of responses, a potential mechanism for change from using the box. Parents identified a number of positive emotions such as confidence and fun, which may have increased their repertoire of playful responses as well as allowing increased attentional resources to be directed towards their children. Crucially as the nature of this theory is cyclical, the broadened responses then in turn increase positive emotions. The same may be true for the infant participants who parents observed displaying a number of positive emotions such as excitement and joy. Frederickson considers that the experience of these positive emotions will also increase individuals’ psychological resources over time. The art boxes could also be seen to facilitate both parent and infant’s ‘Basic Psychological Needs’ as highlighted within self-determination theory (Vansteenkiste et al., 2020). Within this theory wellbeing is fostered when the universal needs of autonomy, competence and relatedness are met. It is clear from the descriptions of families’ experiences how the art boxes could be seen to create conditions conducive to relatedness by bringing dyads, and their wider family group, into rewarding interactions. Similarly there is a clear development of competence, both for the infant who develops mastery of the materials, and the parent who feels increased confidence in ways to play and use materials, as well as their feeling of competence as a parent—as evidenced in their comments about feeling they are doing a good job. Although the activities were guided, the boxes could be seen as providing the opportunity for autonomy; parents were able to draw on our resources to create activity within their day. For the infant, autonomy connects strongly to those signs of agency where infant’s see their own impact on the world in their mark making, which we have highlighted in the themes.

If arts activity can be shown to create positive change for parents-infant dyads, then this could be a valuable tool for improving relationships. This intervention was not one that we had planned, but was an adaption to a very specific set of circumstances. It is very different to what we would offer in ‘normal’ times. We are very mindful of the importance of the therapeutic relationship in art therapy and the containment offered by the art therapist themselves. We also recognise benefits from taking part in a group where there is a collective sense of support from all the families involved and also shared learning. Although a home-based intervention undertaken as a family unit is very different from our usual approach, we have found the results to be surprisingly promising. It is possible that the specific conditions of lockdown, with limited other distractions, actually gave an opportunity when parents were more receptive to this kind of intervention that encourages particular activity with their children. Nonetheless, we are encouraged to reflect on whether there may be potential for this to have benefit which extends beyond the context of lockdown.

One potential criticism is that, by explaining the benefits of art making in our guide, we were potentially predisposing the parents to find them beneficial. However, from our perspective this is a valuable part of the intervention itself, where that element of psychoeducation may act as an important encouragement to take part and see the value for their infant. Indeed, this was highlighted by the parents themselves within the theme of encouragement where parents reported that this push was useful. Another point to note is that we are not able to separate out the different aspects of the intervention, to find a primary mechanism of change. For example, it may be that the difficult feelings which parents related to us (particularly that they had been unsupported during the pandemic), were addressed by the supporting gesture of the box, as much as by the art activity. This does not have to be a negative, as we would see the art box as offering something holistic. This is equally true with traditional art therapy intervention, where there are interconnected benefits from the relationship with the therapist, the social support from the group, the direct psychological intervention, as well as the involvement with the art materials and the creative process. The themes from interviews seem to suggest that there were multiple actions at play for the families from this intervention, although the art process seems to be central in that as a mechanism of bringing dyads into interactions. Potential future research could look at comparing similar interventions using different vehicles for interaction, such as art materials, toys and music.

When thinking of implications for future design we are cautious to suggest that remote intervention, without the presence of in person support, would be sufficient where families present with a high level of need, such as those struggling with mental health difficulties. However, it could certainly be an adjunct to other services. Moreover, we would argue that there is a general need among parents, especially those facing disadvantage, to be supported in building positive relationships with their children. We are aware that there are limited resources within health and social care for supporting families of young children, and for families who do not ‘fit’ referral criteria (e.g., because their problems are systematic rather than related to mental health). Taking a universal approach to encouraging playful interactions using art may be a way to reach families who would not otherwise meet the criteria for support, but who none the less may benefit. The art boxes have shown that with a bit of a nudge to take part, parents were able to see the benefits. This is how we have chosen to take the project forward ourselves, where our next step after this research was to bring in families to help us co-design a book resource on getting started making art with very young children, expanding from the ideas in the art box guide. Our other follow on from the boxes, in 2021 as restrictions continued, was been to use them with voluntary groups in a hybrid format, where we sent the boxes out in advance and then came together to start using the materials in an online session. This was particularly useful with groups where English was not their first language. However, in this case the partner organisation was able to organise a secure internet connection for every participant, without which we would risk excluding some families and heightening disadvantage, so careful consideration is required to use them in this way.

Art seems to be an ideal vehicle for shifting ways in which dyads relate as there are mechanisms for change that are naturally occurring in the process. In which case, how are art organisations able to facilitate more access to art making for families with very young children? It was interesting to observe that while few parents had made art with their children before the intervention and none had used the art venue we are based in. Following the intervention they all continued art making, or planned to do so. This suggests the boxes are a potentially useful way to widen participation and address inequalities of arts access. In turn, the arts in health benefit of such increased involvement is supporting early years development and parental wellbeing by creating opportunities to mechanise positive change within the art process. When interviewees were asked at the end of the interview about their plans to visit the gallery some of their responses highlighted perceived barriers to participation. Although some parents were definite that they wanted to go, some were initially concerned about their infant being too young, or too noisy, or that there may be an associated cost. We were able to explain that the gallery was free and that there were activities specifically for all ages, which seemed to alleviate their concerns. This highlights the need to reach out to families with this kind of information if we want to widen participation. It was a limitation of the study timing that the gallery was actually closed over the interview period due to COVID-19 restrictions, so the information parents shared about plans to visit had to be speculative. It would be useful in future to pair a similar intervention with a follow-up measuring gallery visits to investigate if this did increase attendance.

The last point for discussion is about the usefulness of different kinds of evidence when looking to evaluate an arts-based intervention. Although overwhelmingly positive, we found the quantitative feedback from postcards, that we have included in Supplementary Material, to be of limited utility in understanding more about the mechanisms and impacts of the intervention. In contrast, the analysis of interviews gave an extremely rich picture that was able to describe nuances within the intervention process and detailed observations of parent’s perceptions of changes. However, we acknowledge the limitation of not having baselines to the interviews, or standardised pre- and post-intervention measures which may have allowed us to quantify change. It is also unfortunate that we were unable to make any comparisons with different demographics, such as age, single parent status, levels of deprivation or ethnicity. It is therefore still an open question ‘for whom’ the intervention is effective, although we have a broad picture of the characterisation of our sample. Thus, we would argue that future trialling of this form of intervention might usefully employ mixed methodologies, combining interviews with robust quantitative approaches. Broader quantitative approaches would help us to generalise from the experience of the smaller sample of participants in interviews, and also to address some of the limitations relating to self-selection bias among participants agreeing to be interviewed. Nonetheless, we would argue that gathering the participant voice is a critical first step, often missing in positivist approaches.

## Conclusion

Our findings show parents reporting feeling more confident and undertaking new activities which they plan to continue. This was of particular importance during the 2020 lockdown where parents reported that their opportunities for different experiences became severely limited. Parents described having positive playful interactions using the art materials and reported improvements to their own wellbeing from doing creative activities together with their child. Analysis of these interviews gives a framework for the barriers to connection which they were facing, for how the art box intervention can facilitate moments of connected interaction, and for the impact this can have on the attachment relationship. Our analysis provides promising insights into the mechanism by which this particular scheme has benefitted parent-infant relationships, and suggests that there are attachment benefits from dyadic arts participation. This offers a novel pathway for arts organisations seeking to open up access to art making for disadvantaged families in order to offer arts in health intervention for early relationships.

## Data Availability Statement

The raw data supporting the conclusions of this article will be made available by the authors, without undue reservation.

## Ethics Statement

The studies involving human participants were reviewed and approved by University of Dundee, Schools of Social Sciences and Humanities, School Research Ethics Committee. Our study involved infants in the intervention and the research was conducted with parents. Consent was given by parents for referral and when completing questionnaires (which were optional) and they were provided with a comprehensive participant information sheet. Due to interviews taking place by phone within COVID restrictions, parents provided verbal consent at the outset of the call. As interviews were recorded we have a tangible record of informed consent, fulfilling national and local requirements. Written informed consent was obtained from the individuals’ and/or individuals’ next of kin for the publication of any identifiable data or images included in the manuscript.

## Author Contributions

VA designed and ran the intervention, collected and analysed the data. JR supervised the work and was second coder. All authors contributed to the article and approved the submitted version.

## Funding

The initial stage of this research, delivering art boxes to the first 40 participants, was funded through a University of Dundee, Social Sciences, Phd studentship. The second stage for all subsequent referrals was funded by Wellcome Trust seed funding (grant no. 204816/Z/16/Z), awarded by University of Dundee to the author VA.

## Conflict of Interest

The authors declare that the research was conducted in the absence of any commercial or financial relationships that could be construed as a potential conflict of interest.

## Publisher’s Note

All claims expressed in this article are solely those of the authors and do not necessarily represent those of their affiliated organizations, or those of the publisher, the editors and the reviewers. Any product that may be evaluated in this article, or claim that may be made by its manufacturer, is not guaranteed or endorsed by the publisher.
