# Supplementary material for: The Experiences of Parents and Infants Using a Home-Based Art Intervention Aimed at Improving Wellbeing and Connectedness in Their Relationship
Source: Front Psychol. 2022 May 17;13:732562. doi: 10.3389/fpsyg.2022.732562 (PMC9161640; doi:10.3389/fpsyg.2022.732562)
Supplement: Supplementary file 1 [file Data_Sheet_1.pdf]

Supplementary Data Collected:

In addition to the interviews undertaken in the paper, we also asked parents to return feedback as part of our wider reporting to our organisations and funders. We used stamped addressed postcards in every box, asking participants to agree or disagree with five statements based on what we hoped the boxes could achieve: 'The art box encouraged us to try new activities'; 'The art box increased my confidence to do these kinds of activities with my child'; 'I felt connected to my child while doing these activities'; 'I understand why these art and play activities are beneficial for my child'; 'My own wellbeing increases through doing these activities together'. Parents answered on a Likert scale from 1 (disagree), to 5 (agree). There was also a space for open feedback. If participants did not want to leave home during lockdown to post the cards there was an online version (hosted by Gorilla™ software, Anwyl-Irvine et al., 2019) accessed with a link from the postcard. Both postcards and online feedback were completely anonymous. Although positive, this feedback is limited given that their anonymity precludes any in depth quantitative analysis, but we have included them here as supplementary data to be transparent about our process.

Post card feedback

We received a response rate of 37% (57 responses; 23 of these by postcard and 34 online, and of the online responses four of these were without any data entered, and one only included a written comment without any scaling). We would have liked the response rate to be higher but given the wider conditions of the pandemic and the difficulties families were facing, it is not a surprise. It does however increase the risk that we are seeing response bias where the more positive participants are more likely to have returned their survey.

Results were overwhelmingly positive and showed that parents found benefits in all the areas which we were targeting (see Table 1). Parents reported strong agreement that boxes had encouraged them to try new activities, had increased their confidence, that they felt connected to their child during the art making, that they understood the benefits, and that their own wellbeing increased.

Table 1: Results from scaling questions on postcards and online, showing positive responses

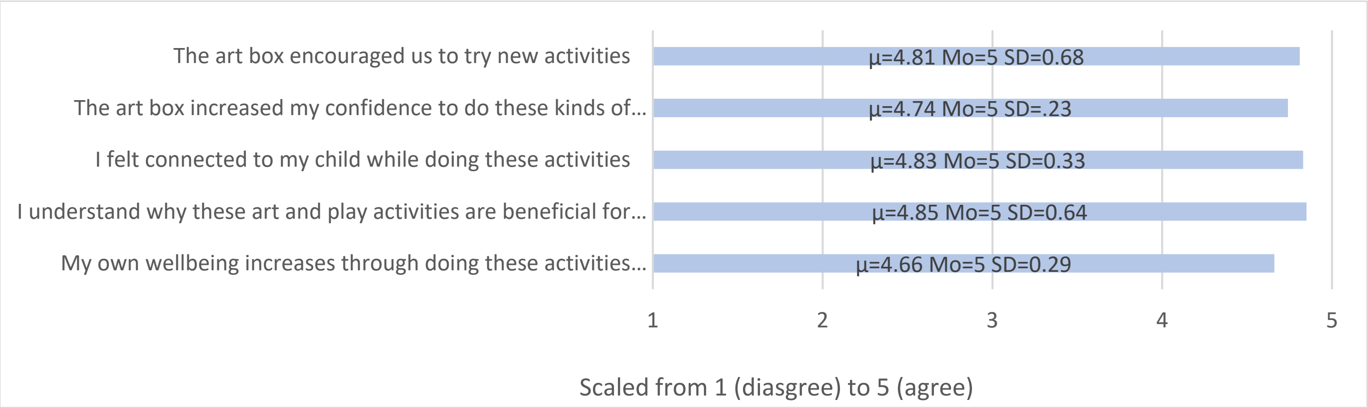

The feedback we received in the open sections of the postcards aligned with the results of the scaling questions. They contained general expressions of gratitude but also comments which told us more about specific changes. Comments identified improvement to parents' mental health (*The box came just at the right time as I had lost my confidence with trying new things and really needed a boost*),

how boxes gave ideas and encouragement (*Easy to follow and things we would not have tried on our own. We have lots left over to keep doing more. Thank you*), how they may have helped encourage parent-infant communication (*helped me communicate and understand my child. I didn't even know my child loves messy play till I got this art pack!*), and how boxes facilitated dedicated time together (*Good to have the time to focus on her and play. Enjoy even to open the box. a gift.*). These topics concur with the more detailed data gathered through interviews as reported in the main paper.

Anwyl-Irvine, A.L., Massonnié, J., Flitton, A. et al. Gorilla in our midst: An online behavioral experiment builder. Behav Res 52, 388–407 (2020). https://doi.org/10.3758/s13428-019-01237-x
