# Supplementary material for: The Experiences of Parents and Infants Using a Home-Based Art Intervention Aimed at Improving Wellbeing and Connectedness in Their Relationship
Source: Front Psychol. 2022 May 17;13:732562. doi: 10.3389/fpsyg.2022.732562 (PMC9161640; doi:10.3389/fpsyg.2022.732562)
Supplement: Supplementary file 2 [file Data_Sheet_2.pdf]

Interview Topic Guide

For parent interviews

Intro:

Thank them for agreeing to speak and explain wat we are doing again - “We are looking to learn a bit more about families experiences of using the boxes together. I know you have received the participant information leaflet in your box so I’m just going to explain again how it will work to check you are still happy.”

Explain process of interview and recording - “I’m going to record the sound from our conversation using Microsoft teams which saves it to the universities internal Onedrive and this doesn’t go outside of the university. I’ll then make a transcription and delete the recording. I will deliberately not use your names or anything when we’re talking, but if you do use a name don’t worry because I will take that out the transcript anyway so it will be totally anonymous.”

Check this is OK and give them a chance to ask questions.

Explain you will press record now and then will ask them to give their consent again so it is recorded and before starting the questions.

Turn on teams

Tell them it’s recording and ask for verbal consent again

Question guide:

- Had you tried making art together before? What kinds? What materials?
- Have you been to DCA with your wee one before?
- What was your experience of finding things to do together in lockdown? (or something re lockdown experiences)
- Were you offered other kinds of support? (or did you feel supported?)
- Did you try the activities in the box together?
- Did anyone else in your family join in?
- Were there any activities that you/your wee one particularly enjoyed? Or didn’t?
- Do you feel confident to do these kinds of activities together?
- Did the activity plans help you or could you do them without these?
- Did you notice any changes in your wee one?
- Did you notice any changes yourself?
- Did you feel making art together helped you to connect with your little one? If not answered above
- And maybe something comparing to other activities e.g. did making art together feel different from other activities you do together?
- Will you keep making art with your wee one?
- Do you think you will come in to DCA together when it opens? for public Messy Play sessions or Family Art Labs or maybe just to look at the exhibition and use the free family room, the Create Space ?

For referrer interviews

Intro:

Thank them for agreeing to speak and explain wat we are doing again - "We are looking to learn a bit more about families experiences of using the boxes together. I know you have received the participant information leaflet in your box so I'm just going to explain again how it will work to check you are still happy."

Explain process of interview and recording - "I'm going to record the sound from our conversation using Microsoft teams which saves it to the universities internal Onedrive and this doesn't go outside of the university. I'll then make a transcription and delete the recording. I will deliberately not use any of your client's names or details when we're talking and stick to speaking generally rather than about specific people, but if anything identifiable slips through I will take that out the transcript anyway so it will be totally anonymous."

Check this is OK and give them a chance to ask questions.

Explain you will press record now and then will ask them give their consent again so it is recorded and before starting the questions.

Turn on teams

Tell them it's recording and ask for verbal consent again

Question guide:

- Had your families engaged with DCA or other arts venues before?
- How do you think the families you support were experiencing lockdown in general?
- Speaking generally, what were the kinds of issues that caused you to refer parents for an art box?
- Were there other options available for support for them to refer to?
- Do you feel the box had an impact on your client/s and their families?
- Did you have any feedback from parents?
- Is this a service that they would use in normal circumstances as well as during covid?
- Do you think this might help to encourage families to engage with the gallery and what's on offer there? (if not already covered above)
